# A Functional Cartography of Cognitive Systems

**DOI:** 10.1371/journal.pcbi.1004533

**Published:** 2015-12-02

**Authors:** Marcelo G. Mattar, Michael W. Cole, Sharon L. Thompson-Schill, Danielle S. Bassett

**Affiliations:** 1 Department of Psychology, University of Pennsylvania, Philadelphia, Pennsylvania, United States of America; 2 Center for Molecular and Behavioral Neuroscience, Rutgers University, Newark, New Jersey, United States of America; 3 Department of Bioengineering, University of Pennsylvania, Philadelphia, Pennsylvania, United States of America; 4 Department of Electrical & Systems Engineering, University of Pennsylvania, Philadelphia, Pennsylvania, United States of America; University of Toronto, CANADA

## Abstract

One of the most remarkable features of the human brain is its ability to adapt rapidly and efficiently to external task demands. Novel and non-routine tasks, for example, are implemented faster than structural connections can be formed. The neural underpinnings of these dynamics are far from understood. Here we develop and apply novel methods in network science to quantify how patterns of functional connectivity between brain regions reconfigure as human subjects perform 64 different tasks. By applying dynamic community detection algorithms, we identify groups of brain regions that form putative functional communities, and we uncover changes in these groups across the 64-task battery. We summarize these reconfiguration patterns by quantifying the probability that two brain regions engage in the same network community (or putative functional module) across tasks. These tools enable us to demonstrate that classically defined cognitive systems—including visual, sensorimotor, auditory, default mode, fronto-parietal, cingulo-opercular and salience systems—engage dynamically in cohesive network communities across tasks. We define the network role that a cognitive system plays in these dynamics along the following two dimensions: (i) stability vs. flexibility and (ii) connected vs. isolated. The role of each system is therefore summarized by how stably that system is recruited over the 64 tasks, and how consistently that system interacts with other systems. Using this cartography, classically defined cognitive systems can be categorized as ephemeral integrators, stable loners, and anything in between. Our results provide a new conceptual framework for understanding the dynamic integration and recruitment of cognitive systems in enabling behavioral adaptability across both task and rest conditions. This work has important implications for understanding cognitive network reconfiguration during different task sets and its relationship to cognitive effort, individual variation in cognitive performance, and fatigue.

## Author Summary

As we go about the day, our brains must quickly adapt in accordance with our internal goals. These modifications generally occur within the constraints of a fixed structural architecture, thus manifesting as rapid changes in the recruitment of and integration between brain regions conforming to task demands. For example, regions of the visual system are preferentially recruited as we perform a visual task, motor regions are recruited in the execution of actions, frontal networks bias signals to other networks by differentially integrating across them, and the default mode network is activated during introspection as opposed to task execution. However, there is as yet no common framework under which the role of each cognitive system in a task can be defined in a normative way with respect to both other systems and the rest of the brain. Here, we address this issue by formalizing a network-based theoretical framework in which the role of each cognitive system is defined based on its level of recruitment and integration defined dynamically across a battery of cognitive tasks.

## Introduction

A major goal of cognitive neuroscience is to discover the role that each brain region plays in enabling complex behaviors [1]. The search for this map between structure and function has advanced significantly over the past few decades [2]. Large volumes of cortical tissue such as the visual or auditory cortex are mapped to gross functions such as sensory perception [3, 4]; while small volumes are mapped to subfunctions, such as the processing of faces and places [5]. Historically, these maps of regions to roles have been defined primarily based on univariate or multivariate neural responses to a variety of task conditions [6]. Recent efforts have extended these tools to encompass the interactions between regions in the context of a functional network spanning the entire brain [7, 8]. However, principled approaches by which whole-brain functional networks can inform the roles of brain regions in enabling behavior remain limited [9].

Network theory offers a framework in which regions or cognitive systems can be compared with one another according to their topology, lending insight into their function [7] and evolution [10]. Indeed, network statistics derived from graph theory have been successful in summarizing the topology of brain regions [11] and systems [12] involved in resting state functions, potentially shedding light on their roles in enabling behavior [13]. For instance, the fronto-cingulo-parietal network is comprised mainly of nodes with extensive inter-system connections, potentially facilitating its role as a control circuit for diverse cognitive processes [12, 14, 15]. While these approaches provide valuable information about average resting state function, they remain unable to address the dynamic roles of cognitive systems elicited by task execution, where systems may integrate with one another in complex and continually changing spatio-temporal patterns [16–19].

Indeed, understanding how cognitive systems interact with one another during different tasks is critical to understand cognitive processing. Evidence suggests that network topology differs in different task states [20–23], and indeed task state can be predicted from patterns of functional connectivity using machine learning techniques [24–28]. Functional network topology also changes with task practice [17, 18, 29–31] and with the degree of cognitive effort required by the task [32]. Fundamental features that characterize these changes are far from understood, but preliminary evidence suggests that interactions between cognitive systems form fundamental drivers for network reconfiguration across task demands [33], facilitating cognitive processes from learning [30] to memory [34]. Yet, a definitive statistical framework in which to characterize and categorize the time-dependent interactions between cognitive systems is lacking.

**Fig 1:**
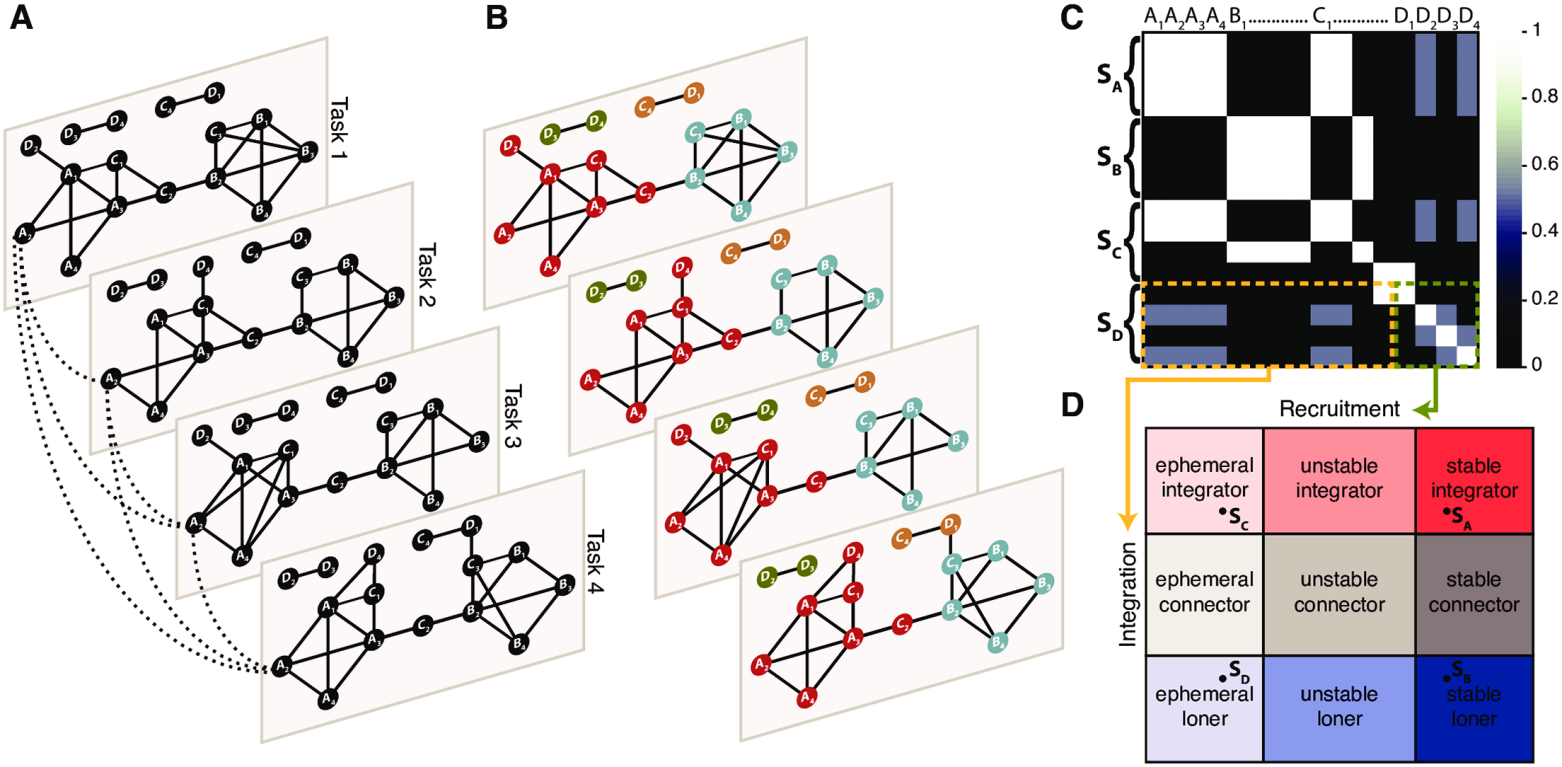
Overview of the methods. (A) A functional connectivity matrix is derived for each task condition as the Pearson correlation between the time series of every pair of nodes. This results in a set of weighted graphs of the same size, one for each task condition. For simplicity, the network is represented here as a binary network, with nodes identified by an index and by an association to a putative system (e.g. *A*_1_, *A*_2_, …). To identify the community structure of this multislice network, the identity of each node is imposed by adding interslice connections (dashed lines) between identical nodes across slices. (B) Using a dynamic network clustering approach known as multislice community detection (see Methods), we extract the community structure of the network for each individual task. Each community is represented by a different color. (C) The module allegiance matrix conveniently summarizes the community structure of the network across tasks. Each entry *i*, *j* of the matrix corresponds to the percentage of tasks in which regions *i* and *j* belong to the same community, describing how regions (*A*_1_, *A*_2_, …, *D*_4_) and large-scale systems (*S*_*A*_, *S*_*B*_, *S*_*C*_, *S*_*D*_) are dynamically engaged during the task battery. Nodes that tend to co-occur in the same communities are represented by brighter colors than nodes that tend to operate in isolation. (D) By comparing the recruitment and integration of each system with a null-model (see Methods), we can extract 9 system-independent ‘network roles’, yielding a cartographic representation of cognitive systems. The systems depicted in the previous insets (*S*_*A*_, *S*_*B*_, *S*_*C*_, *S*_*D*_) represent examples that could occupy each of the four corners.

To address this gap, we make use of the time-dependent community structure of functional brain networks to characterize the dynamic roles of cognitive systems as they evolve during task performance. We derive a set of networks representing functional brain connectivity matrices during different tasks (Fig 1A). We then identify the community structure of the network (Fig 1B), which uncovers functional clusters (communities) composed of groups of brain regions that display dense functional connections with other regions in their group and sparse functional connections with regions in other groups. To characterize the dynamic roles of cognitive systems, we summarize the co-occurrence of regions (or systems) in communities with a module allegiance representation (Fig 1C): pairs of brain regions that co-occur often in the same community, across tasks, have high allegiance values. Finally following [30], we define the *dynamic network recruitment* of a system as the probability that its regions co-occur in communities with regions from the same system, and the *dynamic network integration* as the probability that its regions co-occur in communities with regions from other systems. We combine these two dimensions into a cartographic representation, which classifies cognitive systems into system-independent ‘network roles’ according to their dynamic pattern of intra- and inter-module allegiance over task conditions (Fig 1D), from ephemeral integrators (which transiently integrate with other systems) to stable loners (which remain functionally separated across the entire task battery). The resulting cartography parsimoniously summarizes dynamic roles of cognitive systems in the context of task execution.

We apply this approach to context-dependent functional connectivity matrices extracted from BOLD fMRI data acquired during the performance of 64 cognitive tasks. The tasks were defined using the Permuted Rule Operations (PRO) cognitive paradigm [35], which contains 12 rules that are permuted into 64 distinct but related task states co-localized in short task blocks (see S1 Fig). We hypothesized that brain regions within known cognitive systems would be dynamically recruited consistently across task states. Additionally, we hypothesized that systems would vary in the degree to which they were integrated in the brain: regions in executive systems being more dynamically integrated with other cognitive systems for control and management, and regions in the default mode or cerebellar systems being less dynamically integrated with other cognitive systems. Finally, we hypothesized that the dynamic roles of cognitive systems would vary in rest versus task states: executive systems being less dynamically integrated during rest than during active task conditions.

## Results

### Recruitment of and Integration Between Brain Areas

**Fig 2:**
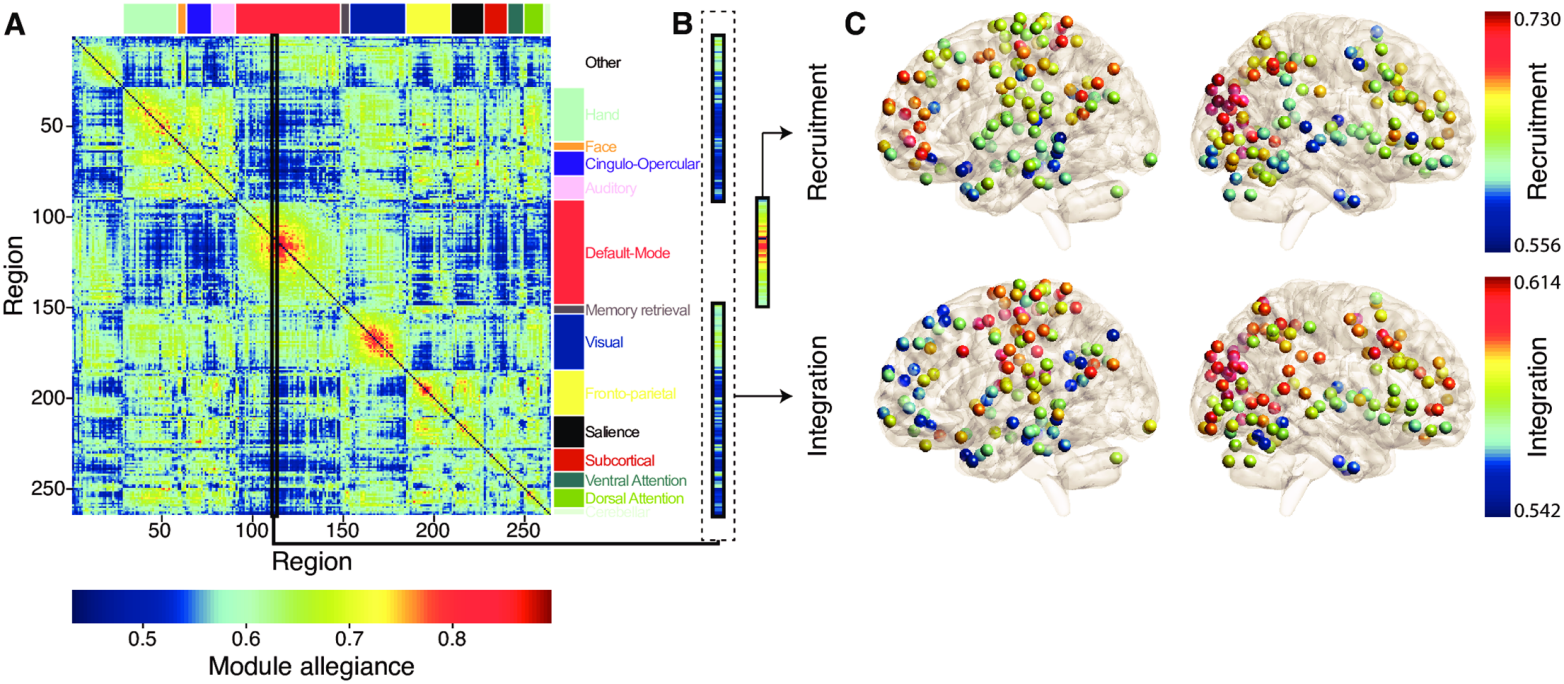
Recruitment and Integration Coefficients Across Brain Regions. (A) The module allegiance matrix represents the probability that two brain regions are part of the same community across the 64-task battery. Here, we ordered the nodes according to which cognitive system they belong to. Note the warm block-like structure along the diagonal of the matrix, which suggests that cognitive systems identified from resting-state data tend to be recruited together during task execution [33]. (B) Within each row/column of the module allegiance matrix, the average allegiance value within a system yields the recruitment coefficient, and the average value outside of a system yields the integration coefficient. Here, we highlight one column of the module allegiance matrix, corresponding to one node within the default mode network, to illustrate the calculation of the recruitment coefficient and the integration coefficient of individual brain regions. (C) Recruitment *(top)* and integration *(bottom)* coefficients for each brain region. Recruitment and integration are weakly correlated over brain areas (Pearson’s correlation coefficient *r* = (262) = 0.31). Note that these measures vary across the brain and that nearby regions tend to have similar recruitment and integration coefficients, potentially revealing the presence of a larger scale structure.

Intuitively, a cognitive system can be defined as a set of brain areas that work together to enable a cognitive function. Pragmatically, a cognitive system can be identified as a set of brain areas that are more functionally connected to other members of the set than to the rest of the brain. Prior work has uncovered 14 such cognitive systems from resting state fMRI data acquired in *N* = 264 brain areas [13] and we hypothesized that these systems would also be recruited cohesively as network communities during task execution (See Supplement for a discussion regarding important considerations associated with this choice of parcellation). To verify our hypothesis, we used a multislice community detection algorithm [36] to identify groups of brain regions that form network communities (putative cognitive systems) in each of 64 distinct but related tasks. We then summarized these data by calculating an *N* × *N* module allegiance matrix, where the *ij* ^th^ element gave the percentage of tasks in which both region *i* and region *j* belonged to the same community. The module allegiance matrix provides a summary representation of how brain regions and large-scale systems are dynamically and cohesively engaged during the task battery (Fig 2A).

The dynamic role of a brain region within this wider network can be parameterized along two dimensions: How cohesively recruited is the region to its own cognitive system across the task battery? And how consistently integrated is the region with other cognitive systems across the task battery? Using the module allegiance matrix, we define two coefficients to address these questions. The *recruitment coefficient* $\left(R_{i}^{S}\right)$ estimates the probability over the task battery that a given region *i* in system *S* is in the same network community as other regions of *its own* system. The *integration coefficient* $\left(I_{i}^{S}\right)$ estimates the probability over the task battery that a given region *i* in system *S* is in the same network community as regions from *other* systems (see Fig 2A and 2B for a schematic representation of the concepts and see Methods for mathematical definitions). Importantly, these two coefficients are not mathematically equivalent to either the strength of functional or structural connectivity emanating from a region. Instead, dynamic network recruitment and integration quantify the time-dependent probability that a region will associate with different systems under different tasks (*integration*) or the time-dependent probability that a region will associate with its own system under different tasks (*recruitment*).

We observe that brain areas differ in their degree of dynamic network recruitment to a cognitive system across the task battery, and in their degree of dynamic network integration with brain regions of other systems (Fig 2C). These differences suggest that regions may play different roles within a cognitive system in the execution of this task battery and accompanying neurophysiological processes. Despite this variability, we also notice a more general trend: that regions tend to be recruited to their own cognitive system, and are less integrated with other cognitive systems, as demonstrated by the warm block-like structures along the diagonal of the matrix shown in Fig 2A. This pattern of results suggests that cognitive systems act as dynamically cohesive network units.

### Roles of Cognitive Systems

The regional results in the previous section tend to support the hypothesis that systems are recruited together as network communities more consistently than non-systems, and are integrated less consistently than non-systems. To address this hypothesis more formally, we define recruitment and integration coefficients for systems as opposed to regions. First, we define the recruitment coefficient of a *system* as the probability that any node from that system is part of the same network community as other regions from that system (see Methods for formal definitions). Similarly, we define the integration coefficient between two systems as the probability that a node from one system is in the same network community as a node from a different system. Using a permutation test in which the associations of regions to systems is permuted uniformly at random (see Methods), we observe that systems tend to be more heavily recruited (*p* < 0.001; $\bar{R}=0.6286$; 95% CI [0.5881, 0.5920]) and less strongly integrated (*p* < 0.001; $\bar{I}=0.5856$; 95% CI [0.5896, 0.5901]) than non-systems, where we define a non-system to be a randomly chosen set of brain areas.

**Fig 3:**
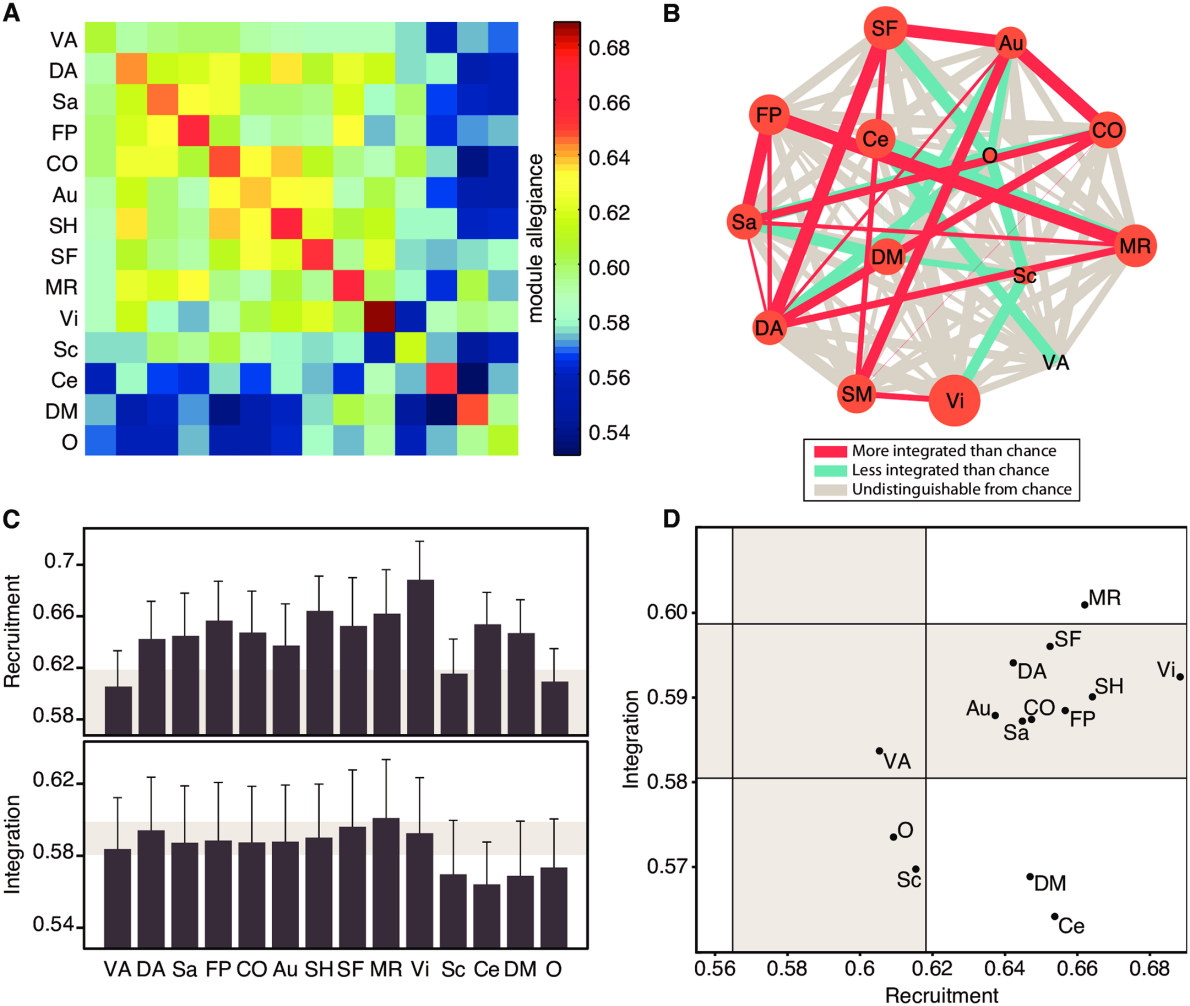
Cognitive Systems Are Differently Recruited and Integrated Across the Task Battery. (A) The module allegiance matrix at the system level represents how regions from large-scale systems are engaged in the dynamic functional brain network during the task battery. Each cell in this matrix is simply the average of the cells within a system, and corresponds to how recruited, on average, are the nodes within a system, and how integrated, on average, are nodes between two given systems. (B) Graph representing the recruitment and integration coefficients of each system. The size of each node represents the recruitment of each system, while the edge thickness represents the integration between a pair of systems. Red edges are significantly stronger than the mean edge weight, and blue edges are significantly weaker than the mean edge weight (*p* < 0.05). (C) Recruitment and integration coefficients. Shaded areas correspond to the range of values expected by a null model, where each brain region is reassigned to a cognitive system uniformly at random. Error bars indicate the standard error of the mean across subjects. (D) Functional cartography of cognitive systems. Each system is represented in a position defined by its average recruitment and integration coefficients. Shaded areas—defined by a null model as in panel (C)—define the significant regions of the parameter space. A specific role is assigned to each of the nine regions of the parameter space, as in Fig 1D. Abbreviations: VA: Ventral Attention; DA: Dorsal Attention; Sa: Salience; FP: Fronto-Parietal; CO: Cingulo-Opercular; Au: Auditory; SH: Somatomotor Hand; SF: Somatomotor Face; MR: Memory Retrieval; Vi: Visual; Sc: Subcortical; Ce: Cerebellar; DM: Default-Mode; O: Other.

While all cognitive systems are consistently recruited as network communities in this 64-task battery, we do observe that systems vary in the strength of dynamic network recruitment and integration (Fig 3A). Some systems—including the visual and somatosensory systems—are consistently highly recruited across the task battery while other systems—including the ventral attention and subcortical systems—are inconsistently recruited across the task battery (see diagonal values in Fig 3A). Furthermore, some systems—including the dorsal attention system—are highly integrated with the majority of other systems, while other systems—including the default mode and cerebellar systems—are weakly integrated with the majority of other systems (see off-diagonal values in Fig 3A).

This type of data can be viewed parsimoniously as a graph, in which nodes represent cognitive systems and edges represent the dynamic network integration between those systems (see Fig 3B). The default mode and cerebellar systems have on average low dynamic network integration with other systems, as does to a lesser extent the subcortical system. We also observe that systems involved in attentional control (cingulo-opercular, dorsal attention, and salience) tend to be well integrated among themselves, as well as with the motor, fronto-parietal, and memory systems. Finally, we see that the fronto-parietal and memory systems are well integrated with one another, consistent with their shared role in executive processing. This graphical representation allows us to see that cognitive systems form a heterogeneous collective, linked by complex dynamical integration patterns over a task battery.

Characterizing cognitive systems in terms of their pairwise integration with other systems can reveal interesting aspects of how different systems of the brain interact in order to allow for complex tasks to be executed efficiently. To summarize the roles of cognitive systems within this broader milieu, we calculated each system’s average dynamic network integration (to all other systems) and dynamic network recruitment during the task battery (Fig 3C). We used these two variables to map cognitive systems into a 2-dimensional interaction space, providing a cartographic representation of task-based cognitive function (Fig 3D). We then assigned a role title to each cognitive system by determining whether its dynamic network recruitment and integration were less than, similar to, or greater than expected in an appropriate null model. In the context of dynamic network recruitment, systems could be classified as either (i) ephemeral (less than null), (ii) unstable (similar to null), or (iii) stable (greater than null); in the context of dynamic network integration, systems could be classified as either (i) loner (less than null), (ii) connector (similar to null), or integrator (greater than null).

In this functional cartography, the memory system is a *stable integrator*, with both high recruitment and high integration coefficients. The default mode and cerebellar systems are *stable loners*, with high recruitment but low integration coefficients. The subcortical system is an *unstable loner*, while most of the remaining systems are *stable connectors*. The ventral attention system was the only system indistinguishable from the null model, receiving the generic label of *unstable connector*.

### Divergent Roles of Cognitive Systems in Distinct Cognitive States

When building a map of the roles that cognitive systems play in the execution of this task battery and associated neurophysiological processes, it is critical to ask whether such a map is generalizable across brain states, or whether it remains specific to the task set chosen. Is the brain characterized by a single map or by a family of maps, each obtained from different sets of cognitive states? In other words, do all cognitive systems always play the same role in every set of tasks, or do they play different roles, and if so, how different? To address these questions, we extracted the functional cartography from resting state data acquired on the same individuals who performed the 64 task battery (see Methods).

**Fig 4:**
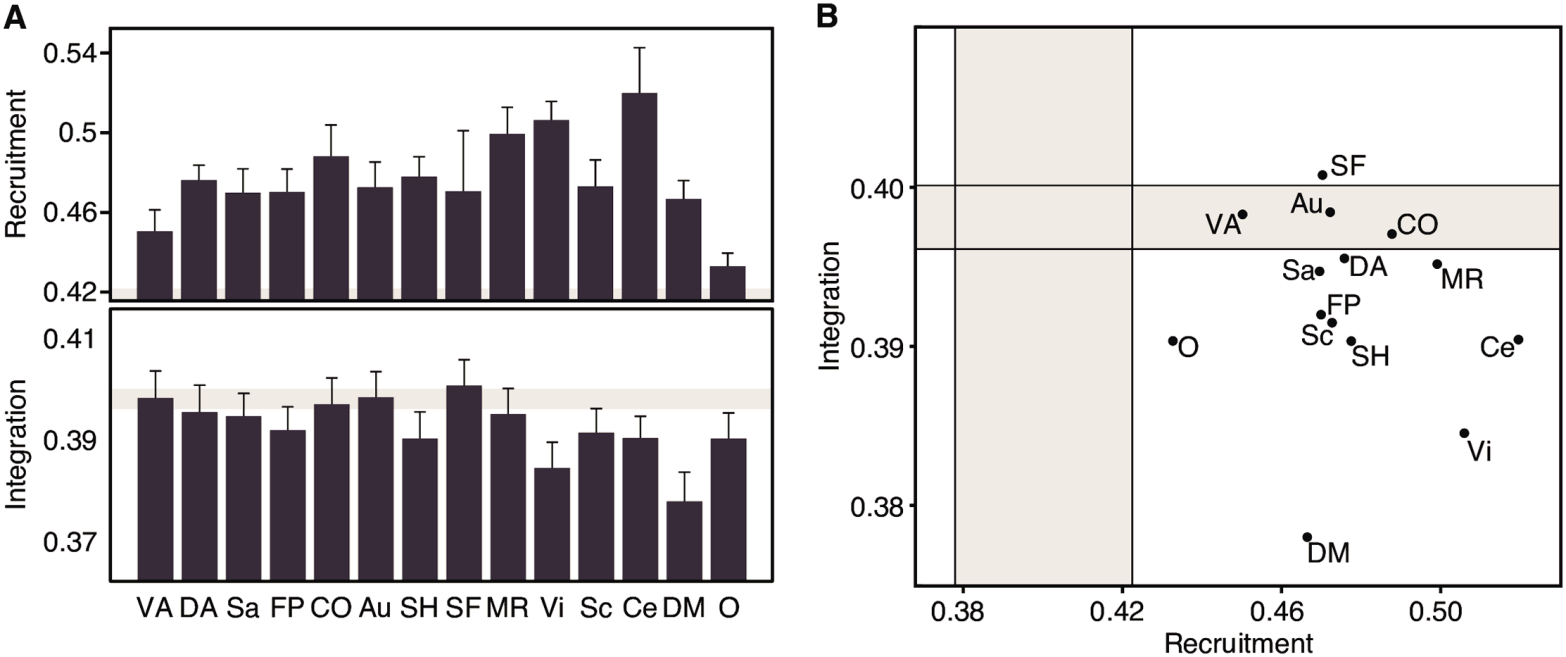
Cognitive Systems Are Differently Recruited and Integrated During the Resting State. (A) Recruitment and integration coefficients for cognitive systems during the resting state. Shaded areas correspond to the range of values expected by a null model, where each brain region is randomly assigned to a cognitive system. Error bars indicate the standard error of the mean across subjects. (B) Functional cartography of cognitive systems in the rest state. Each system is represented in a position defined by its average recruitment and integration coefficients. Shaded areas—defined by a null model as in panel *(A)*—define the significant regions of the parameter space. Abbreviations as in Fig 3: VA: Ventral Attention; DA: Dorsal Attention; Sa: Salience; FP: Fronto-Parietal; CO: Cingulo-Opercular; Au: Auditory; SH: Somatomotor Hand; SF: Somatomotor Face; MR: Memory Retrieval; Vi: Visual; Sc: Subcortical; Ce: Cerebellar; DM: Default-Mode; O: Other.

We observed that the values obtained for the recruitment and integration coefficients reduced to approximately half of that observed in the 64-task data (Fig 4A). The reduced dynamic network recruitment could be caused by a higher temporal variability in rest than task states. We tested this possibility by calculating the network *flexibility*, defined as the proportion of task conditions in which a brain region changes its community allegiance, averaged over all brain regions in the network. Indeed, the average flexibility of subjects was significantly greater in the rest state than in the task state (two-sample *t*-test *t*(14) = 6.24, *p* < 0.001). The reduced dynamic network integration, on the other hand, cannot be understood solely in terms of network flexibility, and suggests that the rest state is a baseline condition of brain functioning, with fewer interactions between systems than is necessary for task execution. Three distinct roles are observed in the rest data: the somatomotor face system as a *stable integrator*; ventral attention, auditory and cingulo-opercular systems as *stable connectors*, and the remaining as *stable loners* (Fig 4B).

**Fig 5:**
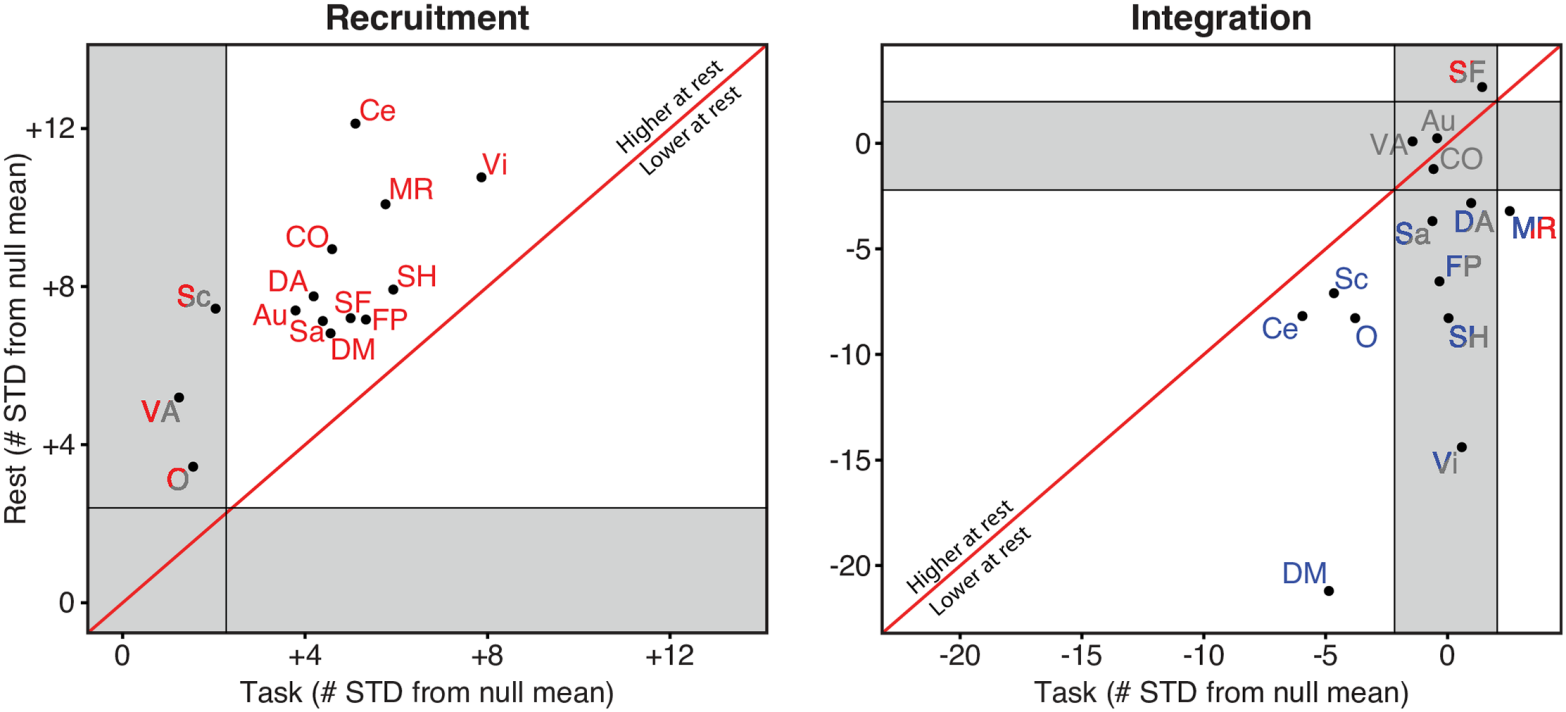
Cognitive Systems Are More Recruited and Less Integrated During Rest. The recruitment and integration coefficients of each system were compared to its task and rest null models. Axes are expressed in terms of number of standard deviations away from the mean of the null distribution. The region above/below the diagonal line indicates that the coefficient is higher/lower at rest than during task. Systems whose coefficients are significantly above the null model are colored in red; systems whose coefficients are significantly below the null model are colored in blue; systems whose coefficients are indistinguishable from the null model are colored in gray. Systems that changed subregions between task and rest are represented in two colors, one for each condition.

A direct contrast between task and rest was achieved by comparing the dynamic network recruitment and integration of each system with its respective null models. This comparison revealed a general trend for cognitive systems to be more highly recruited during rest, indicating an overall greater temporal cohesiveness of each cognitive system—consonant with the fact that these systems were defined from resting state data. In particular, the three systems labeled as *unstable* during task—subcortical, ventral attention and ‘other’—were labeled as *stable* during rest (Fig 5, left panel).

In contrast to the 64-task-based cartography, most of the integration coefficients at rest were lower than the null model, indicating a tendency for cognitive systems to become more temporally dissociated during the rest state. This pattern is particularly salient in the memory system, which changes its role from a *stable integrator* in the task state to a *stable loner* in the rest state. In addition, several other systems labeled as *connectors* during task are classified as *loners* during rest: dorsal attention, salience, fronto-parietal, somatomotor hand and visual. The default mode system serves as another striking example of dynamic dissociation during rest. While it remained a *stable loner* across conditions, its integration coefficient reduced from approximately five to twenty standard deviations below the null model mean, supporting its role as an internally cohesive but externally isolated system (Fig 5, right panel).

## Discussion

The human brain is composed of areas that each perform specialized functions and yet work together in concert to enable behavior. An area’s role in enabling dynamic cognitive computations within this network can be reflected in its profile of functional connectivity to other areas. A rapid and efficient reconfiguration of an area’s connectivity allows for novel and non-routine tasks to be implemented on short time scales. In this study, we presented a novel method to characterize the dynamics of brain network reconfiguration as human subjects performed a battery of 64 distinct but related tasks. We use these methods to assign network roles to different cognitive systems based on (i) how stably the system is recruited over the 64 tasks, or (ii) how consistently the system interacts with other systems. The resulting cartographic representation provides a useful conceptual framework for understanding the unique roles of cognitive systems in enabling behavioral adaptability.

### System-General Features

As the brain flexibly processes the 64 distinct but related tasks, cognitive systems all share two common features: systems are more heavily recruited and less heavily integrated than non-systems. These results suggest that cognitive systems act as cohesive network units within the whole-brain, potentially forming putative functional modules that each enable specific cognitive processes. Such a decomposable organization [37] is conceptually in line with the historical appreciation of “modules” in the brain, which—while not completely encapsulated as suggested several decades ago [38]—form cohesively interacting sets of brain areas that enable a specific cognitive process or behavior.

With the advent of network science, it has become possible to quantify the presence of modules in the brain as groups of regions that are more densely functionally connected than expected in an appropriate null model. The modular organization of the brain, evident in studies of both structural and functional connectivity and across a range of spatial scales [11, 39–41], is thought to enable efficient processing of specialized cognitive functions while maintaining global network integration [42]. Indeed, clinical conditions in which brain network modularity has been altered are commonly associated with broad impairments in cognitive and behavioral abilities. For example, changes in modular structure in schizophrenia [43], unipolar depression [44], autism [45], and bipolar disorder [46] accompany the presence of diverse psychiatric symptoms. Our work complements these studies underscoring the biological significance of functional modules, by examining the temporal stability or flexibility of modular structure as the human brain processes diverse task demands. Our results demonstrate that cognitive systems form network modules, which while locally flexible, are globally stable across disparate cognitive processes. It is intuitively plausible that the balance between local flexibility and global stability might facilitate the adaptive yet robust nature of cognition [18].

When probing the relationship between the brain’s modular architecture and its network dynamics, it is critical to take care in the interpretation of network statistics and to clarify features of system activity and connectivity that may occur at different scales and in different imaging modalities. For example, it is important to distinguish between a brain region’s recruitment to a task as measured by the magnitude of BOLD activity [47] *versus* its dynamic network recruitment over a task battery as measured by the time-dependent probability that the region will associate with its own system under different task conditions [30]. Indeed, patterns of activity and patterns of (dynamic) connectivity offer unique and complementary insights into the neurophysiological processes that accompany and drive cognition [17, 30, 48–50]. Similarly, it is important to note that the dynamic network integration between systems is not mathematically equivalent to the structural or functional connectivity between those systems, and can therefore provide differential sensitivity to individual differences in human behavior [30]. More generally, therefore, it is possible for a system to display strong structural or functional connectivity to other systems but display weak dynamic network integration, indicating a low probability of its regions aligning with the network communities of other cognitive systems during a task battery.

### System-Specific Roles

While cognitive systems share features of high dynamic network recruitment and low dynamic network integration, they also display broad heterogeneity, consistent with their differential roles in the cognitive processes elicited by the 64-task battery. The cartographic representation we propose provides dynamic network roles of cognitive systems that quantify the reconfiguration of system interactions that enable the required cognitive processes (Fig 1D).

#### Stable integrators

The memory system is assigned the role of ‘stable integrator’, being both highly dynamically recruited and highly dynamically integrated. This role is consistent with the system’s anatomical composition: the memory system contains one of the most highly structurally and functionally integrated regions of the brain—the precuneus [51]—along with portions of the posterior cingulate and lateral parietal cortex [13].

#### Stable loners

In contrast, the default mode and cerebellar systems were assigned the role of ‘stable loners’, being highly dynamically recruited but weakly dynamically integrated with other systems. Anatomically, the cerebellar cortex is a parallel-fiber system and this cytoarchitectonic organization promotes little interactions between distant patches [52, 53], potentially leading to its loner personality. The default mode system, on the other hand, is anatomically distributed throughout the cortex and contains both highly connected areas (e.g., precuneus [51, 54]) and highly disconnected areas (e.g., frontal cortex [55]). Functionally, however, the segregation of the default mode system is readily apparent in its classically observed competition with the task-positive network: functional activation of the default mode consistently accompanies functional de-activation of the task-positive network, underscoring its role as a competitive rather than integrative system [56, 57]. In line with these observations, recent work has described the default mode system as a “provincial” hub, given its low inter-system functional connectivity in non-dynamic contexts [13, 58].

#### Unstable systems

The subcortical system is assigned the role of ‘unstable loner’ and the ventral attention system [59] is assigned the role of ‘unstable connector’. The unreliable dynamic network recruitment of these systems across the 64-task battery indicates that these systems do not form temporally cohesive network units. It is likely that the task battery we study here does not recruit the entire set of regions to the same degree as the rest state did in which they were defined.

#### Stable connectors

Finally, the remaining systems were named stable connectors, indicating their roles as temporally cohesive network units with typical levels of dynamic network integration.

### The Default Mode System as a Stable Loner

Perhaps one of the most surprising roles uncovered by this dynamic network analysis is the role of the default mode system as a stable loner. The reason this is surprising is that the default mode system is often thought of as a central system in the brain’s structural connectome. Recent evidence demonstrates that several default mode regions (apart from the inferior parietal lobule, medial temporal, and medial prefrontal cortex) [60–63] are located in the rich-club of white matter architecture [64]. The rich-club [65] is a set of densely interconnected hub nodes, whose connections collectively span the rest of the brain. Why then is the default mode a stable loner in terms of its network dynamics? The implications of structural network organization for task-based functional connectivity and its dynamics are relatively under-explored [66]. Some very recent theoretical, computational, and empirical work has provided initial evidence that this structural connectome can facilitate the coalescence of low-degree nodes into functional subgraphs [67], but that structural connections are not the only (or best) predictor of dynamic functional connections, which can instead be driven by polysynaptic pathways [68] (See also the Supplement for supporting evidence that the default mode system is strongly functionally segregated in these data). It is therefore clear that a comprehensive assessment of the expected relationship between the structural connectome’s rich club, the default mode system, and dynamic network recruitment and integration in task-based analyses is an important direction for future research.

### Roles of Cognitive Systems at Rest

Our results also reveal some fundamental differences in the cartography of cognitive systems between the resting state and the set of states elicited by the 64-task battery. First, we observed greater temporal variability in rest than task states, as evidenced by the fact that both recruitment and integration coefficients reduce to approximately half of their task-based values. This variability can be caused by less stable communities, with regions often changing their community membership. We directly tested this hypothesis by demonstrating a lower average flexibility in the task state than in the rest state. This result is initially counterintuitive, as the task state requires subjects to perform 64 different tasks while the rest state requires subjects to simply stay awake. The apparent paradox can be resolved in light of recent findings that the so-called resting state is, in fact, a collection of many states dynamically fluctuating over time [69–72]. In contrast, the 64 task battery constrains the brain to move within a fixed 64-dimensional space in fixed temporal intervals, potentially leading to the observed low flexibility values.

Second, while most systems were assigned similar roles in both the resting state and the set of states elicited by the 64-task battery, a few systems changed their roles and therefore deserve additional attention. For example the memory system changed from a stable integrator in the task state to a stable loner in the rest state, indicating a large dissociation of this system during rest. Similarly, the visual system became less integrated in the rest state, changing from a stable connector to a stable loner. These findings indicate that visual and memory systems tend to become more isolated from the rest of the network, acting as independent units whose regions have a less-than-average chance of associating with other systems. We speculate that the dissociation of the visual system potentially reveals the rupture of the association between external visual stimuli and internal mental states that was necessary during the task conditions. The dissociation of the memory system in resting state is consistent with the recently observed causal interactions between rest and the central executive system: inhibitory theta-burst transcranial magnetic stimulation to central executive regions enhances default mode activity [73], indicating a causal and competitive relationship between the two systems.

It has been recently suggested that the intrinsic network architecture across tasks is highly similar to the resting-state architecture [33]. Our results complement these findings by showing that the average dynamic network recruitment and integration is substantially different between the resting state and the set of states elicited by the 64-task battery. Specifically, in the task-state, recruitment and integration coefficients are both higher than in the rest-state. This suggests that, on average, cognitive systems are more consistently engaged as temporally cohesive entities, and that they also have more temporally consistent network interactions between one another in the task battery than they do at rest. This is possibly because the types of cognitive processes involved in the tasks are more constrained (and require more energy) than the types of cognitive processes that are present during rest. In contrast, at rest, the most energetically efficient configuration is to have cognitive systems at baseline (i.e. not integrating), and have them be recruited sparingly. This can be understood as a cost-efficient solution to brain activity.

### Implications for Cognition

Cognitive functions are traditionally studied by manipulating distinct task elements and observing differential neural and behavioral responses [74]. To address multiple cognitive states, current techniques combine two tasks together simultaneously [75, 76], or study the transitions between two distinct tasks separated in time (often referred to as “task switching”) [77, 78]. Yet, because the putative task elements are often domain specific, this approach does not lend itself to cross-domain comparisons. Here we offer such an approach by defining the relationship of each region or system to the rest of the brain. This framework allows for different tasks—or entire task sets—to be compared with one another in a statistically principled manner, even when the tasks or task-sets being compared are quite different (See the Supplement, for example, for a contrast between the dynamic network recruitment and integration of cognitive systems in the 64-task set and a 7-task set from the Human Connectome Project). This approach provides a mathematically robust complement to existing correlative methods, which have already proven useful in understanding structural underpinnings of cognitive function [21, 22], the control of cognitive processes [79], and the brain’s dynamic cognitive range [20, 23, 80].

The ability to study task sets begs the question of how tasks are related to one another. The traditional view of mental chronectomy from Donders (1969) [81] suggests that brain states are additive and therefore separable in both space and time. This assumption, which fundamentally underlies cognitive neuroscience, supports the notion that one can identify and compare cognitive processes using linear (difference or subtraction) methods [82]: one can take one cognitive process and compare a second process that differs in only one important respect, and conclude that the difference between the two cognitive processes is revealed in the difference between the two sets of measurements associated with them [83]. Critically, this approach enables one to study supposed individual task elements, even if these elements can never be experienced in isolation. However, mounting evidence suggests the need to nuance this so-called “process isolation” approach, by developing methods that can account for nonlinear dependencies between cognitive processes [83]. For example, it has been shown that some cognitive control areas are differentially activated by switches between different sets of tasks [78], in different contextual variations [84], with different rewards [85], with skill acquisition [86], and in switching from task A to task B versus switching from task B to task A [87–89]. The existence of these non-additive effects complement known nonlinear processes identified in healthy [83] and diseased [90] cortical activity.

However, it remains an open question how the brain moves between larger sets of tasks in a time-dependent fashion. What network reconfigurations are required for a given trajectory through the set of tasks? Do some trajectories or some task sets require different types of reconfiguration patterns? Are there higher order temporal dependencies between network reconfigurations required for consecutive task switches? Initial evidence from the literature suggests that such higher order relationships do in fact exist. These studies are consistent with the notion that cognitive systems dynamically adapt their network properties in a task-**set**-specific (as opposed to a task-specific) manner, and therefore motivate the development of novel methods to quantify these reconfiguration properties. The functional cartography approach that we develop and apply here provides a quantitative characterization of the dynamic network role of cognitive systems as the brain transitions between multiple tasks. In the future, these tools could be used to quantify the cognitive difficulty of a set of tasks, or a trajectory through a set of tasks, by determining distributions of recruitment and integration across cognitive systems. Additionally, linking these quantifications of network reconfiguration to individual differences in cognitive performance or fatigue during the performance of a set of tasks will be of particular interest.

### Methodological Considerations

Studies of network topology in the brain have historically operated under the implicit assumption of constant interdependence between brain areas. This assumption is evident in the common consideration of a single time window over which measures of functional connectivity such as correlation or coherence are computed and presumed to be constant. While these approaches have provided us with a great understanding of large-scale properties of brain function, alternative formulations that consider the dynamic nature of these interdependence measures can reveal spatially distributed regions whose interdependencies change from time to time or from condition to condition [17, 18, 71, 72, 91]. In particular, the modular organization of the resting state network is itself time-dependent, being altered, for instance, by cognitive effort [92] or learning [17]. Here we present an approach that directly addresses the dynamic interaction of large-scale brain networks involved in cognitive processing. By summarizing these dynamic interactions in the form of dynamic network recruitment and dynamic network integration, we can extract functional roles of both regions and systems in any set of brain state conditions (e.g., temporal conditions or task conditions).

It is important to define what the term *dynamic network integration* means in our formulation, particularly as it distinguishes reliable partnering between systems from a random re-coupling. A system with a high integration coefficient value must not only exhibit frequent repartnering over time, but must also display at least one partner that occurs more often than expected by chance, indicating temporal reliability. This is different than what would be expected if nodes were assigned to communities uniformly at random, which would lead to a relatively low average integration coefficient. Indeed, our definition of module allegiance resembles that of a soft-partitioning—which describes the degree or probability of participation of a node to each module [93], generalized to multislice networks.

A second important consideration lies in the fact that our proposed method can be adapted to any spatial scale in which interdependence measures between network nodes can be defined. For instance, dynamic network recruitment and integration can be defined for functionally defined regions (e.g. the fusiform face area [94] or the parahippocampal place area [95] within visual cortex), or anatomically defined regions (e.g. Broadman areas [96]). Moreover, these statistics could be defined for (i) individual neuronal spikes, (ii) LFPs from neuronal ensembles, (iii) EEG or MEG sensors, or (iv) localized sources of EEG or MEG activity.

Finally, the methods and approaches that we introduce and exercise here demonstrate that brain regions can move between communities as task demands change, consistent with prior work in other task contexts [17, 18, 29, 30]. Yet, these observations have been made in the context of fixed definitions for functional nodes and for functional systems. This area is ripe for the development of other complementary approaches that may contribute time- and task-dependent definitions of both nodes and systems.

### Conclusions

The functional cartography of cognitive systems provides a new conceptual framework for understanding the dynamic network integration and recruitment of cognitive systems in enabling behavior, both during task and rest conditions. These methods allow for several avenues of future inquiry, such as the investigation of the cartography for different task batteries or at different spatial and temporal scales, as well as the investigation of individual variability and its utility in understanding behavioral differences in healthy and patient populations.

## Methods

### Ethics Statement

The protocol was approved by the University of Pittsburgh Institutional Review Board (IRB #0702094). All subjects gave written informed consent before the start of the experiment.

### Participants

Fifteen participants (eight male, seven female) ranging in age from 19 to 29 years old (mean = 22 years), voluntarily participated in this experiment. Participants were recruited from the University of Pittsburgh and the surrounding area, and were excluded if they (i) had any medical, neurological or psychiatric illness, (ii) had other contra-indications for MRI scans, (iii) were non-native English speakers, or (iv) were left-handed. Results from unrelated analyses of these data are reported elsewhere [24, 33].

### Task Procedure

Subjects performed the permuted rule operations (PRO) task procedure [24, 33, 35], with task instructions presented using E-Prime software [97]. This procedure combines three task factors, each with four possible levels, yielding a total of 64 distinct but related task combinations (See Supplementary Information and S1 Fig for additional details).

In addition to ten experimental task runs (each 216 TRs in length), each scan session also included one resting state scan (300 TRs in length), during which subjects were instructed to keep their eyes open and focus on a fixation point on the screen.

### MRI Data Collection

Magnetic resonance images were obtained at 3.0-T on a Siemens Trio MRI scanner. T1-weighted structural images were collected using Siemens implementation of GRAPPA, in order to double image acquisition speed. During experimental runs, blood-oxygenation level dependent (BOLD) functional images were collected using an echo-planar pulse sequence (time repetition [TR] = 2000 ms, echo time [TE] = 30 ms, flip angle = 90, voxel size = 3.2 mm isotropic). Functional data were acquired at a field of view of 210 mm across 39 transaxial slices.

### fMRI Preprocessing

Preprocessing of functional data was performed using AFNI [98] and Freesurfer [99], and resting-state and task-state data were preprocessed identically in order to facilitate their comparison. Sinc-interpolation in time was performed to correct for the slice acquisition order, and motion correction using least squares minimization was performed to correct for head motion in the scanner. The resulting functional images were normalized to a Talairach template. Nuisance time series were removed (motion, ventricle and white matter signals, and their temporal derivatives) using a General Linear Model, and spatial smoothing was performed in the gray matter mask (6mm full width at half maximum). The average time series over the entire brain (global signal) also had its contribution removed from the time series of each region, in an effort to increase tissue sensitivity and reduce dependencies on motion [100]. However, given current controversy in the literature [101], we also performed our analyses without global signal regression and observed qualitatively similar results (see Supplementary Materials). Finally, Freesurfer was used to identify ventricle, white matter and gray matter anatomical structures for each participant.

### Functional Connectivity Estimation

Further analysis was carried out using custom code in MATLAB 2012b. In order to reduce the dimensionality of the data and allow inferences at the region and systems levels, voxel-wise functional data was resampled on a set of 264 brain regions comprising the cerebrum and cerebellum. The boundaries of the regions were defined using a combination of resting-state functional connectivity parcellation [102] and task fMRI meta-analyses [13, 103] (See Supplement for considerations regarding this choice of parcellation). The time course of each region was obtained by averaging the signal from all voxels falling inside the region. The entire brain network was also further subdivided at the system-level (subgraphs), where regions within a system are more strongly connected to one another than to the rest of the network [13] (see subsection entitled “Recruitment and Integration” below).

For the 64 task data, we regressed out each task event to suppress influences of shared stimulus-locked activation on the estimation of functional connectivity. Specifically, we estimated context-dependent connectivity using a linear model equivalent to the general linear model (GLM) typically used in fMRI analysis, with one regressor for each task condition convolved with a subject-specific hemodynamic response function. The residuals of this GLM were then used for the estimation of functional connectivity, which involved the calculation of the Pearson product-moment correlation between residuals from each brain region, separately for each task condition. These correlation coefficients were then Fisher-*z*-transformed to improve normality (See Supplement for an alternative method of removing task-effects using a Finite Impulse Response model).

To keep the analyses of resting-state and task-state data consistent, the resting-state time-series from each region was parsed into blocks of 11 TRs (the same approximate duration of a mini-block in the task data), yielding 27 non-overlapping time bins. The Pearson product-moment correlation was calculated between the time-series from each brain region separately for each time bin, and the correlation coefficients were Fisher-*z*-transformed to improve normality (See Supplement for a discussion on the choice of window length).

The procedure was repeated for each subject and for each task condition (time-bin), yielding, for each subject, a set of 64 task-state (and 27 rest-state) 264 × 264 functional connectivity (or adjacency) matrices.

### Multislice Community Detection

Common approaches to data clustering in networks are based on community detection techniques [104, 105]. Here we use a generalized Louvain method [106] for optimizing modularity [107] developed specifically for community detection in multislice systems [36]: systems in which multiple networks (in our case drawn from task blocks) are to be examined at once. For each set of FC matrices from each subject, we implemented a categorical multislice modularity maximization [36, 108] which considers the multiple adjacency matrices as slices of a single network, and imposes consistent node identity across slices by adding interslice connections between each node and itself in every slice of the network. We then optimize the multislice modularity quality Function (1), which uses the relative densities of intra-community connections versus inter-community connections to identify a partition of network nodes into communities or modules [36], and which can be defined as:

$$Q_{\text{multislice}}=\frac{1}{2\mu }\;\sum_{ijsr}\left[\;\left(A_{ijs}-\gamma_{s}V_{ijs}\right)\delta_{sr}+\delta_{ij}\omega_{jsr}\right]\delta \left(g_{is},g_{jr}\right)$$ (1)

where the adjacency matrix of slice *s* has components *A*_*ijs*_, the element *V*_*ijs*_ gives the components of the corresponding matrix for a null model, *γ*_*s*_ is the structural resolution parameter of slice *s*, the quantity *g*_*is*_ gives the community (i.e., “module”) assignment of node *i* in slice *s*, the quantity *g*_*jr*_ gives the community assignment of node *j* in slice *r*, the parameter *ω*_*jsr*_ is the connection strength between node *j* in slice *s* and node *j* in slice *r*, the total edge weight in the network is $\mu =\frac{1}{2}\sum_{jr}\;\kappa_{jr}$, the strength of node *j* in slice *s* is *κ*_*js*_ = *k*_*js*_ + *c*_*js*_, the intraslice strength of node *j* in slice *s* is *k*_*j*_ *s*, and the interslice strength of node *j* in slice *s* is *c*_*js*_ = ∑_*r*_ *ω*_*jsr*_. We employ the Newman-Girvan null model within each layer by using

$$V_{ijs}=\frac{k_{is}k_{js}}{2m_{s}}$$ (2)

where $m_{s}=\frac{1}{2}\sum_{ij}\;A_{ijs}$ is the total edge weight in slice *s*. The free-parameters are the structural resolution parameters, *γ*_*s*_, and the interslice coupling parameters, *ω*_*jsr*_, here assumed to be constant (*γ*_*s*_ = *γ*, ∀*s* and *ω*_*jsr*_ = *ω*, ∀*j* and ∀*s* ≠ *r*, meaning that node *j* in slice *s* connects to node *j* in every slice *r* ≠ *s* with weight *ω*). Respectively, these parameters control the size of communities within a given layer and the number of communities discovered across layers. To choose values for these parameters, we explored the parameter space by running 100 optimizations for each combination of *γ* and *ω*, with both parameters varying between 0 and 10 in intervals of 0.05. The specific parameters used in the main analyses (*γ* = 1 and *ω* = 0.45) were chosen so as to maximize the variability of the flexibility coefficient across brain regions (see Supplementary Information and S2–S4 Figs for a summary of the parameter exploration results). As in previous work [17, 18], we define flexibility of a brain region as the proportion of task conditions in which this region changes its community allegiance. The rationale for using this approach was to enhance differences between nodes with regards to their degree of stability in community allegiance, yielding brain regions that vary maximally between highly flexible and highly stable.

The multislice community detection approach is a crucial part of our description of dynamic roles of cognitive systems. It provides a data-driven clustering of regions into putative cognitive systems that is more statistically robust than examining community structure at individual times or in individual task windows, and provides sensitivity to a wide range of temporal scales of importance in neuroimaging data. This multislice community detection procedure yielded, for each subject, a set of 100 partitions for each of the 64 task (and 27 rest) conditions. In each so-called ‘hard’ partition, each of the 264 nodes is assigned to precisely one community.

### Recruitment and Integration

The multislice community detection procedure reduces the dimensionality of each subject’s data from 264 × 264 × 64 to 264 × 64, where each brain region is assigned to one community in each task condition. The dimensionality of the data can be further reduced by calculating a *module allegiance matrix* *P*, a data-driven summary of the region-to-region (or node-to-node) interactions across the entire task set. The entries of the module allegiance matrix, *P*_*ij*_, were calculated as the percentage of tasks in which the pair of nodes *i*, *j* co-occur in the same community Eq (3):

$$P_{ij}=\frac{1}{OT}\sum_{o=1}^{O}\sum_{t=1}^{T}a_{i,j}^{k,o}$$ (3)

where *O* is the number of optimizations (chosen to equal 100 in this study), *T* is the number of slices (64 task slices or 27 rest slices), and, for each optimization *o* and slice *t*,

$$a_{i,j}^{k,o}=\left\{\begin{array}{cc} 1 & \;\text{if}\;\text{nodes}\;\text{i}\;\text{and}\;\text{j}\;\text{are}\;\text{in}\;\text{the}\;\text{same}\;\text{community}\\ 0 & \;\text{otherwise} \end{array}\right.$$

We use the module allegiance matrix to assess the dynamic roles of cognitive systems during task execution. We first define cognitive systems based on dense subgraphs previously identified from resting-state data [13]. To quantify the dynamic role of a region within one of these systems, we use the module allegiance matrix to compute two coefficients: the dynamic network recruitment and the dynamic network integration. The *recruitment* coefficient of region *i* with respect to system *S*, $R_{i}^{S}$, is defined as:

$$R_{i}^{S}=\frac{1}{n_{S}}\sum_{j\in S}P_{ij}$$ (4)

where *n*_*S*_ is the size of system *S*, calculated as the number of regions in *S*. In other words, $R_{i}^{S}$ corresponds to the average probability that the *i* ^th^ brain region is in the same community as other regions of the system *S*. A region with high recruitment to system *S* is one that tends to be found in system *S* across many task conditions.

The *integration* coefficient of region *i* with respect to system *S*, $I_{i}^{S}$, is defined as:

$$I_{i}^{S}=\frac{1}{N-n_{S}}\sum_{j\notin S}P_{ij}$$ (5)

where *N* is the total number of brain regions. In other words, $I_{i}^{S}$ corresponds to the average probability that the *i* ^th^ brain region is in the same community as regions from systems other than *S*. A region in system *S* with high integration is one that tends to be found in systems other than its own across many task conditions.

### Functional Cartography

While understanding the role of specific regions within the brain is useful, a coarser understanding of the roles of whole systems could provide compelling insights into how system interactions are orchestrated during task execution. We therefore extend the definition of the recruitment coefficient to an entire cognitive system by averaging the recruitment coefficients for all regions within a system *S*:

$$R_{S}=\frac{1}{n_{S}^{2}}\sum_{i\in S}\sum_{j\in S}P_{ij}.$$ (6)

A system that is highly recruited is one whose regions all tend to be placed in the same community throughout the full task battery. Similarly, we extend the definition of the integration coefficient to the system level by computing the average integration coefficient between system *S*_*k*_ and system *S*_*l*_:

$$I_{S_{k}S_{l}}=\frac{1}{n_{S_{k}}n_{S_{l}}}\sum_{i\in S_{k}}\sum_{j\in S_{l}}P_{ij}$$ (7)

which can be viewed as the degree to which system *S*_*k*_ recruits regions from system *S*_*l*_.

Finally, the integration of a system *S* to all other systems is simply the average of the individual pair-wise integrations:

$$I_{S}=\frac{1}{n_{S}\left(N-n_{S}\right)}\sum_{i\in S}\sum_{j\notin S}P_{ij}$$ (8)

where *N* is the total number of brain regions. A system that is highly temporally integrated is one whose regions tend to be placed in communities composed of regions from other systems across the task battery.

Given that the recruitment and integration coefficients are only weakly correlated at the regional level (*r* = (262) = 0.31) and not significantly correlated at the systems level (*r* = 0.45, *p* = 0.11), these two quantities provide complimentary information about cognitive systems. We therefore use these two quantities in concert to provide a description of a system’s dynamic role in the brain’s evolving functional network enabling cognitive performance; see Fig 1D. This functional cartography is a representation that places each cognitive system on a 2-dimensional plane whose dimensions correspond respectively to dynamic network recruitment and dynamic network integration.

To interpret a system’s roles within this cartographic representation, we compare a system’s placement to that expected in an appropriate null model that randomly permutes the correspondence between node and system observed in the real data. In the null model, pseudo-systems (or non-systems) are formed as collections of nodes in the brain chosen uniformly at random. More specifically, we created null partitions for each subject and task block that were random permutations of the region-to-system assignments. This procedure ensured that the number of systems and the sizes of systems in the null model were kept identical to those observed in the real data.

A comparison of each coefficient value to its 95% confidence interval obtained from the null model allows the identification of systems that have a lower, higher or indistinguishable dynamic network recruitment and integration to a non-system. Thus, network roles can be attributed to systems based on this comparison. Cognitive systems are classified as *ephemeral* if they are less dynamically recruited (below 95% confidence interval), *unstable* if they are similarly dynamically recruited (within 95% confidence interval), or *stable* if they are more dynamically recruited (above 95% confidence interval) than the null model. Similarly, systems are classified as *loners* if they are less dynamically integrated, *connectors* if they are similarly dynamically integrated, or *integrators* if they are more dynamically integrated than the null model. An illustration of all nine possible roles is shown in Fig 1D. (For a discussion regarding the system definitions used here, see Supplement).

## Supporting Information

S1 Text Supplementary Methods. (PDF)

S2 Text Supplementary Results. (PDF)

S3 Text Supplementary Discussion. (PDF)

S1 Table Abbreviations. List of abbreviations for cognitive systems
(PDF)

S1 Fig The permuted rule operations behavioral procedure. The permuted rule operations (PRO) procedure allowed for behavioral tasks to be created by uniquely combining rules such that the same stimuli could elicit a distinct set of cognitive operations across distinct tasks. Each one of the 64 tasks combine one of four possible logical decision rules, one of four possible sensory semantic rules, and one of four possible motor response rules. Out of the 64 possible tasks, subjects practiced four in a behavioral session prior to the neuroimaging session and the remaining 60 tasks were practiced for the first time during the scan. Participants were over 90% accurate for both novel and practiced tasks.
(JPG)

S2 Fig Grid search over parameter space. (A) Average and standard deviation of the flexibility coefficient, average community structure similarity across partitions and across tasks, average number of communities, and average partition quality, calculated for structural resolution parameters (*γ*) and interslice coupling parameters (*ω*) varying between 1 and 10 in intervals of 1.0. The optimal combination of parameters is one in which the standard deviation of the flexibility coefficient is maximum, with relatively high community structure similarity across partitions and low community structure similarity across tasks. (B) Average and standard deviation of the flexibility coefficient, average community structure similarity across partitions and across tasks, average number of communities, and average partition quality, calculated for structural resolution parameter (*γ*) and interslice coupling parameter (*ω*) varying between 0.0 and 1.0 in intervals of 0.05.
(PDF)

S3 Fig Robustness of the task-based cartography representation over small variations of parameters. Two-dimensional cartography representations of 14 cognitive systems in the integration-recruitment plane for the following parameter pair choices: (*ω* = 0.45, *γ* = 1), top left; (*ω* = 0.45, *γ* = 0.95), top right; (*ω* = 0.44, *γ* = 1.0), bottom left; (*ω* = 0.46, *γ* = 1.0), bottom right.
(PDF)

S4 Fig Robustness of the resting state cartography representation over small variations of parameters. Two-dimensional cartography representations of 14 cognitive systems in the integration-recruitment plane for the following parameter pair choices: (*ω* = 0.45, *γ* = 1), top left; (*ω* = 0.45, *γ* = 0.95), top right; (*ω* = 0.44, *γ* = 1.0), bottom left; (*ω* = 0.46, *γ* = 1.0), bottom right.
(PDF)

S5 Fig Replication of Fig 3 without global signal regression. (A) Recruitment and integration coefficients for cognitive systems in the resting state. Shaded areas correspond to the range of values expected by a null model, where each brain region is assigned uniformly at random to a cognitive system. Error bars indicate the standard error of the mean across subjects. (B) Functional cartography of cognitive systems in the resting state. Each system is represented in a position defined by its average recruitment and integration coefficients. Shaded areas—defined by a null model as in panel *(A)*—define the significant regions of the parameter space. Abbreviations: VA: Ventral Attention; DA: Dorsal Attention; Sa: Salience; FP: Fronto-Parietal; CO: Cingulo-Opercular; Au: Auditory; SH: Somatomotor Hand; SF: Somatomotor Face; MR: Memory Retrieval; Vi: Visual; Sc: Subcortical; Ce: Cerebellar; DM: Default-Mode; O: Other.
(PDF)

S6 Fig Examining network organization over longer time windows. *(A)* The functional cartography during task execution utilizing a 64-layer network with each layer corresponding to an individual task (see main manuscript). Each system is represented in a position defined by its average recruitment and integration coeffcients. Shaded areas—defined by a null model as in panel *(A)*—define the significant regions of the parameter space. *(B)* Functional cartography during task execution utilizing a reduced, 16-layer network, with each layer corresponding to a group of four tasks with equal sensory and motor rules. Abbreviations: VA: Ventral Attention; DA: Dorsal Attention; Sa: Salience; FP: Fronto-Parietal; CO: Cingulo-Opercular; Au: Auditory; SH: Somatomotor Hand; SF: Somatomotor Face; MR: Memory Retrieval; Vi: Visual; Sc: Subcortical; Ce: Cerebellar; DM: Default-Mode; O: Other.
(PDF)

S7 Fig Grid search over parameter space for the HCP 7-task data set. Parameter search in the Human Connectome Project data set. Average and standard deviation of the flexibility, average *z*-score of partition similarity across multilayer modularity maximization and across tasks, average number of communities, and average partition quality *Q*, calculated for values of the structural resolution parameter (*γ*) and interslice coupling parameter (*ω*) that vary between 0 and 1 in intervals of 0.1. We define the optimal combination of parameters as one in which the standard deviation of the flexibility is maximal, with relatively high *z*-score of partition similarity across multilayer modularity maximization and low *z*-score of partition similarity across tasks.
(PDF)

S8 Fig Task-Based Cartography in the Independent HCP 7-task Data Set. *(A)* Recruitment and integration coefficients for cognitive systems in the Human Connectome Project data set. Shaded areas correspond to the range of values expected by a null model, where each brain region is assigned uniformly at random to a cognitive system. Error bars indicate the standard error of the mean across subjects. *(B)* Functional cartography of cognitive systems in the Human Connectome Project data set. Each system is represented in a position defined by its average recruitment and integration coefficients. Shaded areas—defined by a null model as in panel *(A)*—define the significant regions of the parameter space. Abbreviations: VA: Ventral Attention; DA: Dorsal Attention; Sa: Salience; FP: Fronto-Parietal; CO: Cingulo-Opercular; Au: Auditory; SH: Somatomotor Hand; SF: Somatomotor Face; MR: Memory Retrieval; Vi: Visual; Sc: Subcortical; Ce: Cerebellar; DM: Default-Mode; O: Other.
(PDF)

S9 Fig Removal of Task Effects Through a Finite Impulse Response model. *(A)* The functional cartography during PRO-task execution with a subject-specific HRF estimated and applied to remove residual task-effects prior to the calculation of functional connectivity. We then utilized a 64-layer network with each layer corresponding to the functional connectivity estimated in an individual task (see main manuscript). Each system is represented in a position defined by its average recruitment and integration coefficients. Shaded areas define the significant regions of the parameter space in comparison to a null model. *(B)* Functional cartography during task execution with a Finite Impulse Response model applied to remove residual task-effects. This approach aims to remove task effects by modeling the Hemodynamic Response Function (HRF) independently at each brain region and separately for each task, allowing for different shapes (amplitude and timing) of HRFs. The resulting cartography is obtained from the dynamic functional connectivity analysis performed on the residuals of a General Linear Model including nine regressors for each of the 12 task rules, for a total of 108 regressors. Abbreviations: VA: Ventral Attention; DA: Dorsal Attention; Sa: Salience; FP: Fronto-Parietal; CO: Cingulo-Opercular; Au: Auditory; SH: Somatomotor Hand; SF: Somatomotor Face; MR: Memory Retrieval; Vi: Visual; Sc: Subcortical; Ce: Cerebellar; DM: Default-Mode; O: Other.
(PDF)
