# Supplementary material for: A Functional Cartography of Cognitive Systems
Source: PLoS Comput Biol. 2015 Dec 2;11(12):e1004533. doi: 10.1371/journal.pcbi.1004533 (PMC4668064; doi:10.1371/journal.pcbi.1004533)
Supplement: S1 Text — (PDF) [file pcbi.1004533.s001.pdf]

S1 Text. Supplementary Methods

Task Paradigm

Subjects performed the permuted rule operations (PRO) cognitive paradigm [1], which combines a set of rule components in various possible ways. Four logical decision, four sensory semantic and four motor response rules were used in the procedure. Each task consisted of one rule from each of these categories, allowing the creation of 64 ($4 \times 4 \times 4$) distinct but related tasks by permuting the possible rules. Of these 64 tasks, four (counterbalanced across participants) were practiced (30 blocks, 90 trials each) during a 2-hr behavioral session 1–7 days before the neuroimaging session, and the remaining 60 were executed for the first time during the neuroimaging session. The practiced tasks were chosen for each subject such that each rule was included in exactly one of the four tasks, ensuring that all rules were equally practiced. This also ensured that rule identity was controlled for across novel and practiced tasks (that is, only rule combinations differed across the conditions). During the neuroimaging session, half of the mini-blocks consisted of the practiced tasks and half of novel tasks, randomly interleaved. With ten runs total per participant, each novel task was presented in one mini-block and each practiced task was presented in 15 mini-blocks.

The semantic rules consisted of sensory semantic decisions (for example, Is it sweet?). The logical decision rules specified how to respond based on the semantic decision outcome(s) for each trial (for example, "Are the two items the same in sweetness?"). The motor response rules specified which finger to use to respond based on the logical decision outcome. The task instructions made explicit reference to the motor response for a 'true' outcome (for example, right index finger), and participants knew from the practice session to use the other finger on the same hand (for example, right middle finger) for a 'false' outcome (Fig. S1).

Task mini-blocks included instruction encoding and three trials. Each mini-block began with a task type cue, indicating whether the upcoming task was novel (thin border) or practiced (thick border), followed by three instruction screens. The order of the instructions following the task type cue was consistent for each participant, but counterbalanced across participants. Asterisks filled in extra spaces in each instruction screen to control for differences in total visual stimulation across task rules. Each stimulus was presented for 800 ms with a 200 ms inter-stimulus interval. Inter-event intervals (that is, between instructions and each of the three trials) were randomly varied between 2 and 6 seconds in duration, whereas inter-mini-block intervals randomly varied between 12 and 16 seconds in duration. There were 12 mini-blocks per run (each of approximately 11 TRs in length), with six novel task mini-blocks and six practiced task mini-blocks each. All task mini-blocks were included in all analyses, as there was at least one accurate trial per mini-block for all participants.

Each subject ($n = 15$) performed a set of 64 distinct tasks, composed of unique combinations of task rules [1]. The combinations always involved one logical rule, one sensory semantic rule, and one motor response rule, each one selected out of four possible rules in each category. Each subject practiced four out of the 64 possible tasks prior to the scan session, including one instance of each of the 12 rules. The remaining 60 tasks were executed for the first time during the scan session.

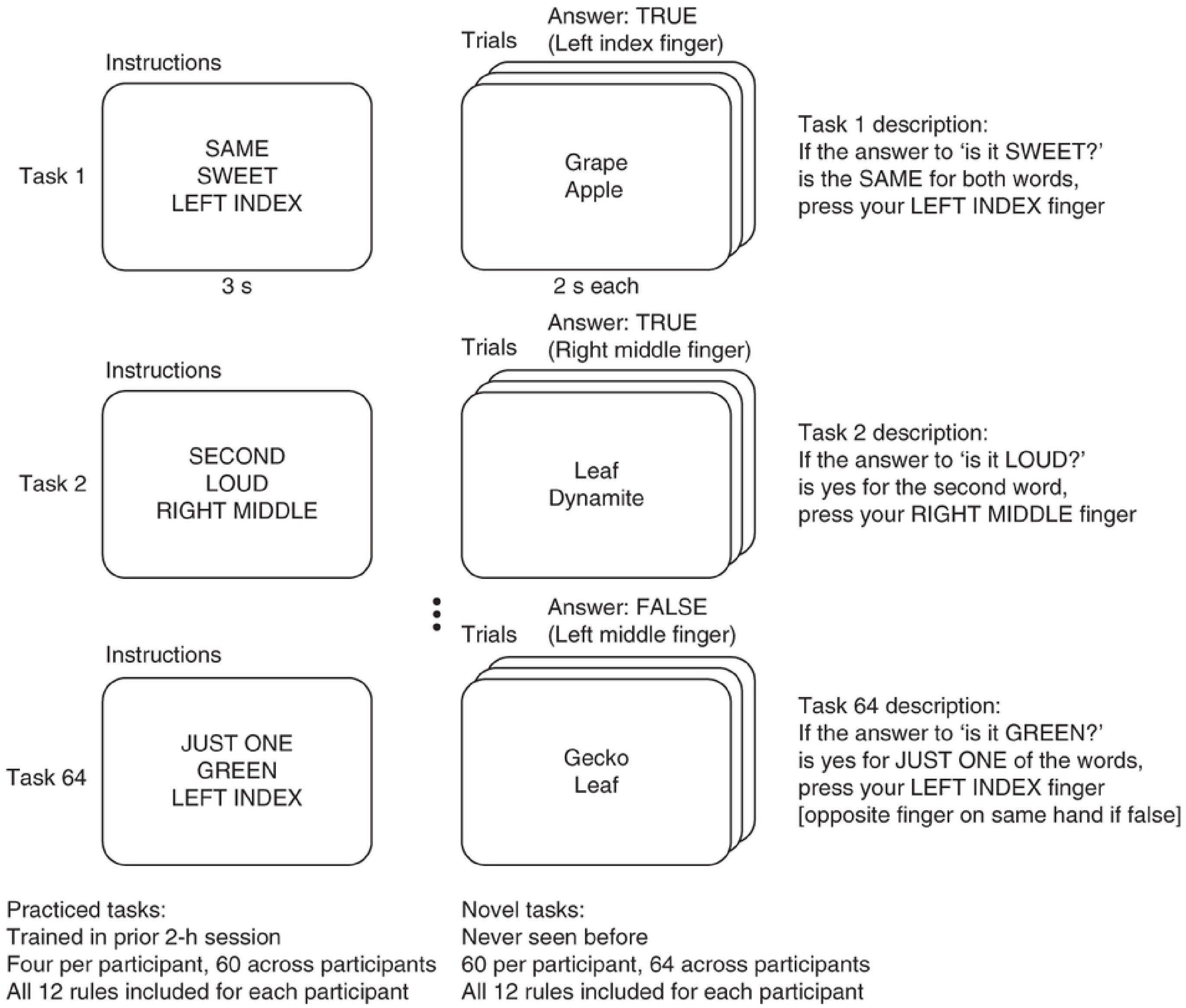

Figure S1: **The permuted rule operations behavioral procedure.**
The permuted rule operations (PRO) procedure allowed for behavioral tasks to be created by uniquely combining rules such that the same stimuli could elicit a distinct set of cognitive operations across distinct tasks. Each one of the 64 tasks combine one of four possible logical decision rules, one of four possible sensory semantic rules, and one of four possible motor response rules. Out of the 64 possible tasks, subjects practiced four in a behavioral session prior to the neuroimaging session and the remaining 60 tasks were practiced for the first time during the scan. Participants were over 90% accurate for both novel and practiced tasks.

Parameter Search

The free parameters of the multislice community detection algorithm – the structural resolution parameter γ, and the interslice coupling parameter, ω – must be selected prior to optimizing the modularity quality function. We tuned these parameters by running a grid-search procedure aimed at maximizing the variability in the flexibility of brain regions. The rationale was that this procedure would avoid parameter combinations where nodes never change communities (since all nodes would have zero flexibility), as well as parameters where nodes always change communites (all nodes would have unit flexibility, and therefore zero variance). In addition, this procedure prioritizes parameter combinations that maximally differentiate between brain regions in terms of their dynamics. A search through the parameter space revealed the parameters $\gamma = 1.0$ and $omega = 0.45$ to yield a high variability in the flexibility coefficient ($SD = 0.0182$) with relatively stable communities (Fig. S2).

In addition to the (i) average and (ii) standard deviation of the flexibility coefficient, we extracted, for each parameter combination, the (iii) average community structure similarity across partitions (as measured by the z-score of the Rand coefficient [2]; the (iv) average community structure similarity across tasks; the (v) average number of communities and; the (vi) average modularity index of the partition.

The structural resolution parameter and inter-slice coupling parameters were first optimized for the task data so as to maximize the across-region variance in inter-task flexibility. We then used the same parameter values for the resting state data, for which the parameter optimization criteria (inter-task flexibility) was undefined. This choice facilitated direct comparison between the task and rest data sets, and further ensured that differences between task and rest were due to differences in dynamic network reconfiguration, and not due to differences in model parameter choices.

Application of Cartographic Methods to HCP Data

Here in the supplement, we examined the task-based cartography derived from an independent task-based fMRI data set collected as part of the Washington University-Minnesota Consortium Human Connectome Project [3]. Participants were recruited from Washington University (St. Louis, MO) and the surrounding area. All participants gave informed consent. The data used were from the first and second quarter releases, consisting of data from 139 participants. Data from 21 subjects were not used because one or more of the data runs were not collected for these subjects, such that data from 118 subjects were included in the analyses. Whole-brain echo-planar imaging acquisitions were acquired with a 32 channel head coil on a modified 3T Siemens Skyra with time to repetition (TR) = 720 ms, time to echo = 33.1 ms, flip angle = 52 degrees, bandwidth = 2,290 Hz/pixel, in-plane field of view = 208 $times$ 180 mm, 72 slices, and 2.0 mm isotropic voxels, with a multiband acceleration factor of 8 [4]. Data were collected over 2 days. On each day 28 min of rest (eyes open with fixation) fMRI data across two runs were collected (56 min total), followed by 30 min of task fMRI data collection (60 min total). Each of the seven tasks was completed over two consecutive fMRI runs. Additional task data collection details for this data set can be found [5].

We sought to preprocess the seven-task data set in a similar manner as the 64-task data set, although some

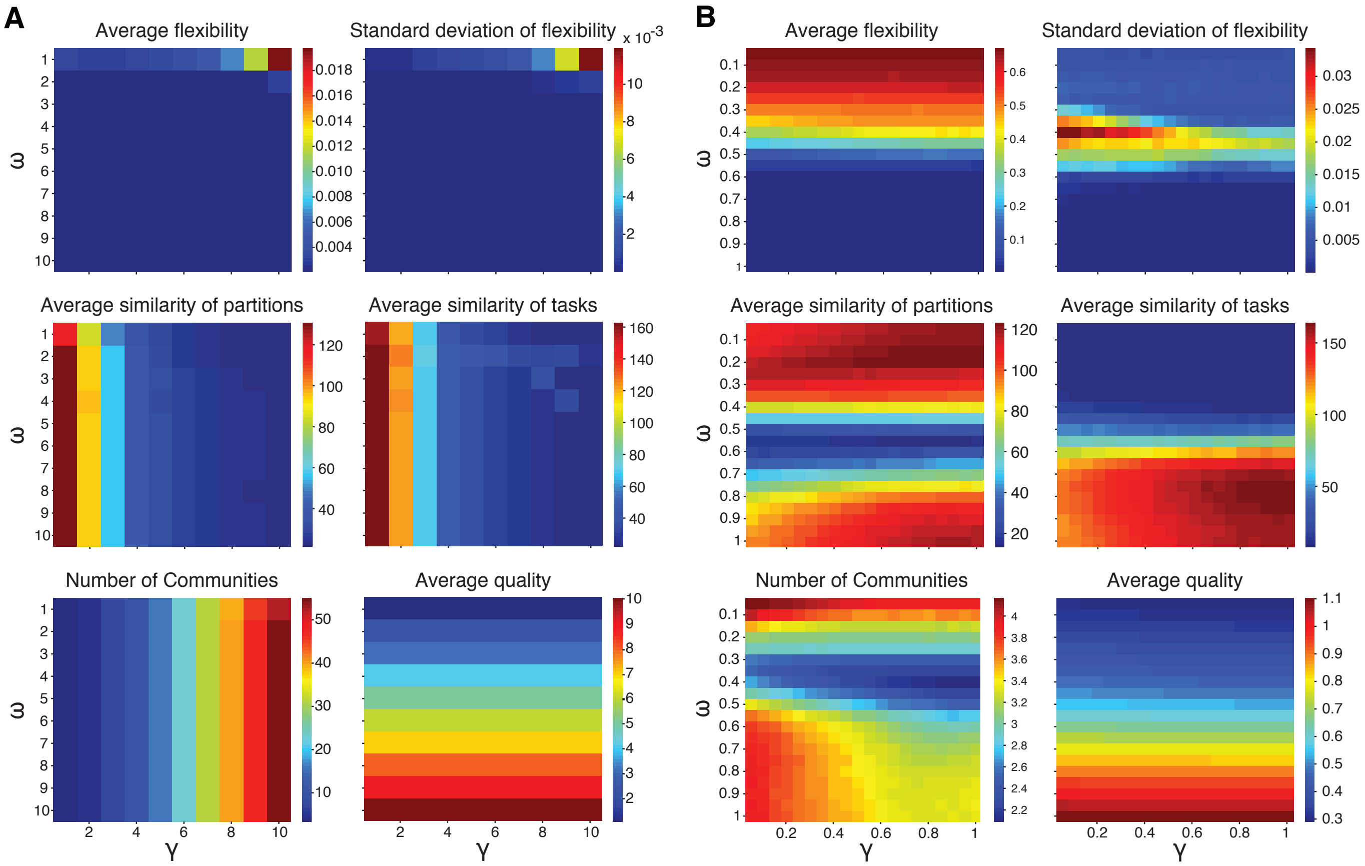

Figure S2: **Grid search over parameter space.**
(A) Average and standard deviation of the flexibility coefficient, average community structure similarity across partitions and across tasks, average number of communities, and average partition quality, calculated for structural resolution parameters (γ) and interslice coupling parameters (ω) varying between 1 and 10 in intervals of 1.0. The optimal combination of parameters is one in which the standard deviation of the flexibility coefficient is maximum, with relatively high community structure similarity across partitions and low community structure similarity across tasks.
(B) Average and standard deviation of the flexibility coefficient, average community structure similarity across partitions and across tasks, average number of communities, and average partition quality, calculated for structural resolution parameter (γ) and interslice coupling parameter (ω) varying between 0.0 and 1.0 in intervals of 0.05.

differences were necessary due to differences in data collection methods. For instance, nonlinear warping was required to correct spatial distortions in this data set. This and related corrections (spatial normalization to a template, motion correction, intensity normalization) were already implemented in a minimally processed version of the seven-task data set described elsewhere [6]. With the volume (rather than the surface) version of the minimally preprocessed data, we used AFNI [7] to additionally remove nuisance time series (motion, ventricle, whole-brain, and white matter signals, along with their derivatives) using linear regression, remove the linear trend for each run, and spatially smooth the data (4 mm full width at half maximum). Unlike the 64-task data set, motion censoring was not applied given relatively minimal movement by participants and a desire to see whether replication of results would be possible without motion censoring. In order to make this data set comparable to the 64-task data set, the data were temporally downsampled (as the last step of preprocessing) by averaging data from every three consecutive volumes (making a 2,160 ms TR, close to the 2,000 ms TR in the 64-task data set). This had an effect similar to a mild low-pass temporal filter on the

data (removing frequencies above 0.46 Hz).

References

[1] Cole, M. W., Bagic, A., Kass, R. & Schneider, W. Prefrontal dynamics underlying rapid instructed task learning reverse with practice. *The Journal of neuroscience* **30**, 14245–14254 (2010).

[2] Traud, A. L., Kelsic, E. D., Mucha, P. J. & Porter, M. A. Comparing community structure to characteristics in online collegiate social networks. *SIAM Review* **53**, 526–543 (2011).

[3] Van Essen, D. C. *et al.* The WU-Minn Human Connectome Project: an overview. *Neuroimage* **80**, 62–79 (2013).

[4] Uğurbil, K. *et al.* Pushing spatial and temporal resolution for functional and diffusion MRI in the Human Connectome Project. *Neuroimage* **80**, 80–104 (2013).

[5] Barch, D. M. *et al.* Function in the human connectome: task-fMRI and individual differences in behavior. *Neuroimage* **80**, 169–189 (2013).

[6] Glasser, M. F. *et al.* The minimal preprocessing pipelines for the Human Connectome Project. *Neuroimage* **80**, 105–124 (2013).

[7] Cox, R. W. AFNI: software for analysis and visualization of functional magnetic resonance neuroimages. *Comput. Biomed. Res.* **29** (1996).
