# Supplementary material for: A Functional Cartography of Cognitive Systems
Source: PLoS Comput Biol. 2015 Dec 2;11(12):e1004533. doi: 10.1371/journal.pcbi.1004533 (PMC4668064; doi:10.1371/journal.pcbi.1004533)
Supplement: S2 Text — (PDF) [file pcbi.1004533.s002.pdf]

S2 Text. Supplementary Results

Robustness Over Parameter Variation

The results from the parameter exploration revealed that basic properties of the community structure obtained for the networks depend on the selection of the free parameters, as expected mathematically. Not surprisingly, these parameters also impact the overall cartography of cognitive systems because the relevant spatial scales are tuned by the structural resolution parameter (γ) and the temporal stability across tasks is tuned by the interslice coupling parameter (ω). Despite this innate and mathematically expected variability over the parameter space, it is also important to demonstrate that the results reported in the main manuscript are not unique, and instead can be robustly identified in a range of parameter values. We therefore investigated how the features of the cartographic representation change over the range $\omega \in [0.44, 0.46]$ and $\gamma \in [0.95, 1.0]$. We observed that the overall structure of the task-based (Fig. S3) and resting state (Fig. S4) cartography remained relatively constant over this parameter range, indicating robustness of the cartography representations reported in the main manuscript.

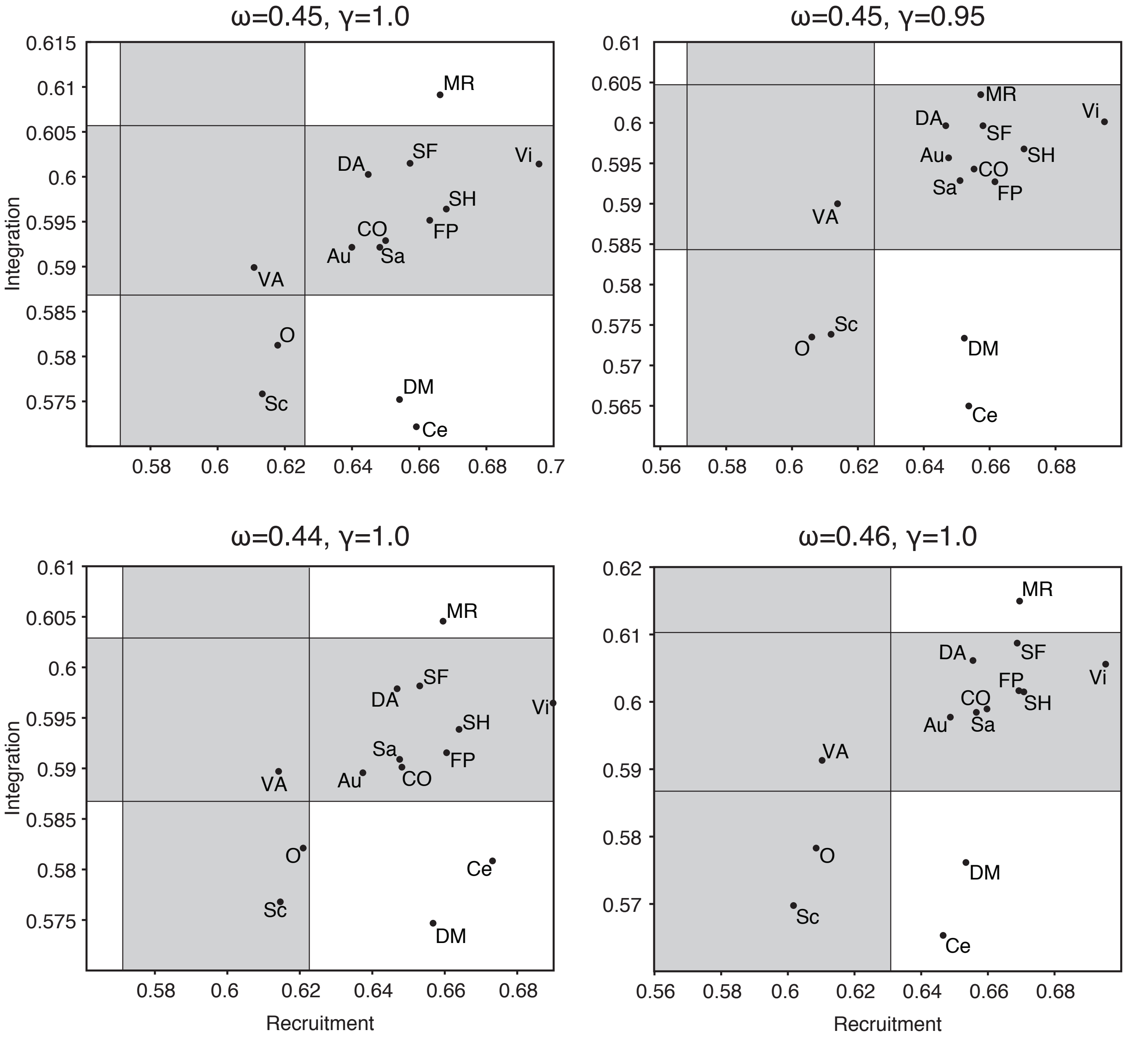

Figure S3: **Robustness of the task-based cartography representation over small variations of parameters**

Two-dimensional cartography representations of 14 cognitive systems in the integration-recruitment plane for the following parameter pair choices: ($\omega = 0.45$, $\gamma = 1$), top left; ($\omega = 0.45$, $\gamma = 0.95$), top right; ($\omega = 0.44$, $\gamma = 1.0$), bottom left; ($\omega = 0.46$, $\gamma = 1.0$), bottom right.

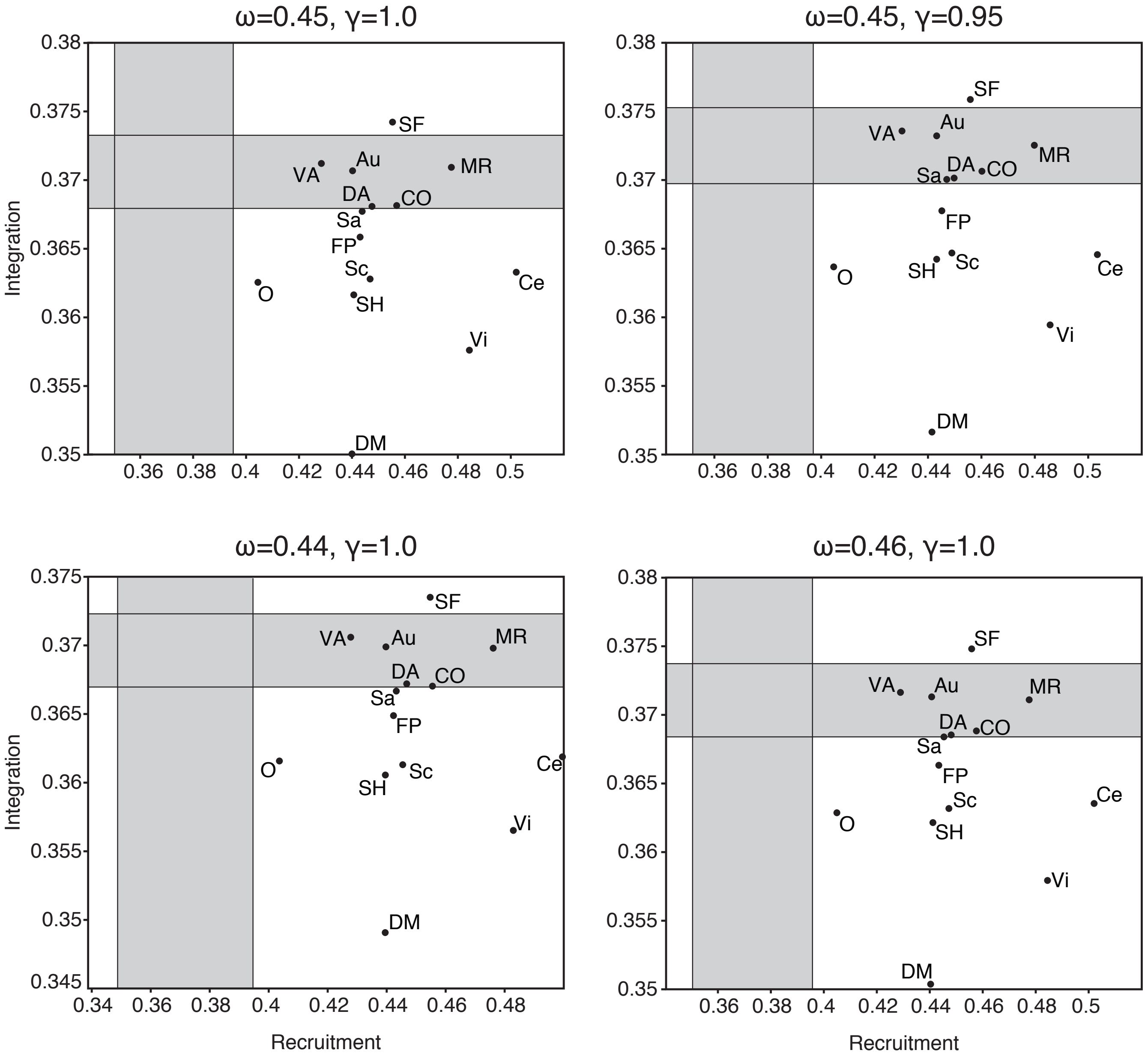

Figure S4: **Robustness of the resting state cartography representation over small variations of parameters**

Two-dimensional cartography representations of 14 cognitive systems in the integration-recruitment plane for the following parameter pair choices: ($\omega = 0.45$, $\gamma = 1$), top left; ($\omega = 0.45$, $\gamma = 0.95$), top right; ($\omega = 0.44$, $\gamma = 1.0$), bottom left; ($\omega = 0.46$, $\gamma = 1.0$), bottom right.

Effect of Global Signal Regression

Any analyses that involve the calculation of correlations between time-series can be influenced by the presence of a strong global signal component. To account for these nuisance effects, a commonly used approach in preprocessing the BOLD signal is to regress out the global signal. However, given that this approach enforces the distribution of correlation values to have zero mean, it can in some instances induce artifactual negative correlations[1].

In the main text of this paper, we presented results from a set of analyses that included global signal regression, in an attempt to increase tissue sensitivity and reduce dependencies on motion [2]. However, given the controversy over this approach, we conducted the same analyses without global signal regression (Fig. S5). Our results show the robustness of our method to global signal regression, as evidenced by the similarity of the results. This is likely a consequence of the community detection approach, which compares the correlation values within and across communities to a null model, and not to a pre-determined baseline (e.g. correlation zero).

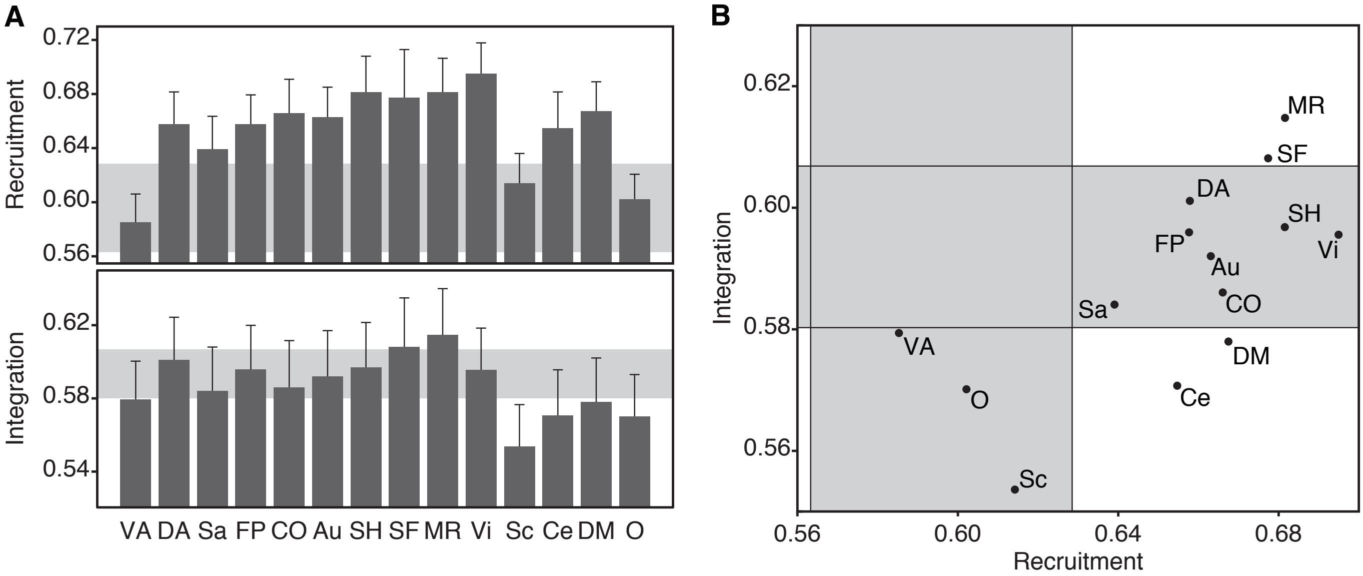

Figure S5: **Replication of Fig. ?? without global signal regression.**
(A) Recruitment and integration coefficients for cognitive systems in the resting state. Shaded areas correspond to the range of values expected by a null model, where each brain region is assigned uniformly at random to a cognitive system. Error bars indicate the standard error of the mean across subjects.
(B) Functional cartography of cognitive systems in the resting state. Each system is represented in a position defined by its average recruitment and integration coefficients. Shaded areas – defined by a null model as in panel *(A)* – define the significant regions of the parameter space.
Abbreviations: VA: Ventral Attention; DA: Dorsal Attention; Sa: Salience; FP: Fronto-Parietal; CO: Cingulo-Opercular; Au: Auditory; SH: Somatomotor Hand; SF: Somatomotor Face; MR: Memory Retrieval; Vi: Visual; Sc: Subcortical; Ce: Cerebellar; DM: Default-Mode; O: Other.

Functional Segregation of Default Mode from Other Systems

Given the role of the default mode system as a stable loner, it is interesting to ask whether this system is strongly or weakly functionally connected to other systems in the 64-task data. To address this question, we follow [3] and define the *within-system connectivity* as the average strength of functional connections between regions in the same system,

and we define the *between-system connectivity* as the average strength of functional connections between regions in one system and regions in another system. We note that these definitions are independent of any community detection, but are simply performed on the functional connectivity matrices themselves. We observe that connectivity within the default mode system is significantly greater than connectivity from the default mode system to other systems: a two-sample t-test of within- versus between-system connectivity gave $t = 13.98$, $p = 1.3 \times 10^{-9}$. Critically, connectivity between the default mode system and the rest of the brain (mean between-system connectivity was -0.022, STD $= 0.0077$) was weaker than the connectivity between any other system and the rest of the brain (minimum between-system connectivity was -0.013, STD= 0.0049). These results indicate that in fact the default mode system is strongly functionally segregated in this data, consistent with its low values of dynamic network integration. These observations are also consistent with other studies in independent adult and youth samples, which describe the default mode system as a "provincial" hub, given its low inter-system functional connectivity in non-dynamic contexts [4, 5].

Examining network organization over longer time windows

As in any dynamic network analysis, it is important to consider the effect of time window length. In the main text, we treated each of the 64 tasks as the minimum temporal unit, ensuring an assessment of brain connectivity during each separate task. However, it remains of interest to investigate the effect of using longer time windows, which will offer a tradeoff of increased statistical power in estimating functional connectivity and decreased power to assess task-specific functional connectivity patterns. To quantify these effects, we combined time segments from all four logical decision rules into a single time-series, yielding a total of 16 individual tasks (combining four sensory semantic rules and four motor response rules). We then calculated the pairwise functional connectivity between all pairs of nodes and proceeded with the analysis in a fashion identical to that reported in the main manuscript. We observed that the overall profile of the resulting cartography is visually distinct from that obtained from single-task windows. Specifically, all systems occupy only two out of nine possible quadrants, indicating a significant decrease in the dynamic range of network roles played by cognitive systems. This reduced role range, together with an overall decreased integration coefficient, are a direct consequence of the insensitivity to functional connectivity patterns characteristic of single task states.

Task-Based Cartography in the Independent HCP 7-task Data Set

In the main manuscript, we demonstrate that the cartographic mapping of the human brain depends on the set of states that the brain moves through: the set of brain states elicited in the 64-task data set produce a distinct cartography from the set of states elicited in the resting state data set. Yet, it remains unclear whether the cartography observed in the 64-task data set is representative of task batteries more generally. To address this gap, we applied the analysis pipeline to an independent sample drawn from the Human Connectome Project (HCP). This sample consisted of task-based fMRI data acquired from 100 subjects performing 7 highly distinct tasks referred to as emotional, gambling, language, motor, relational, social, and N-back. Data were acquired over the course of two 30-minute scanning

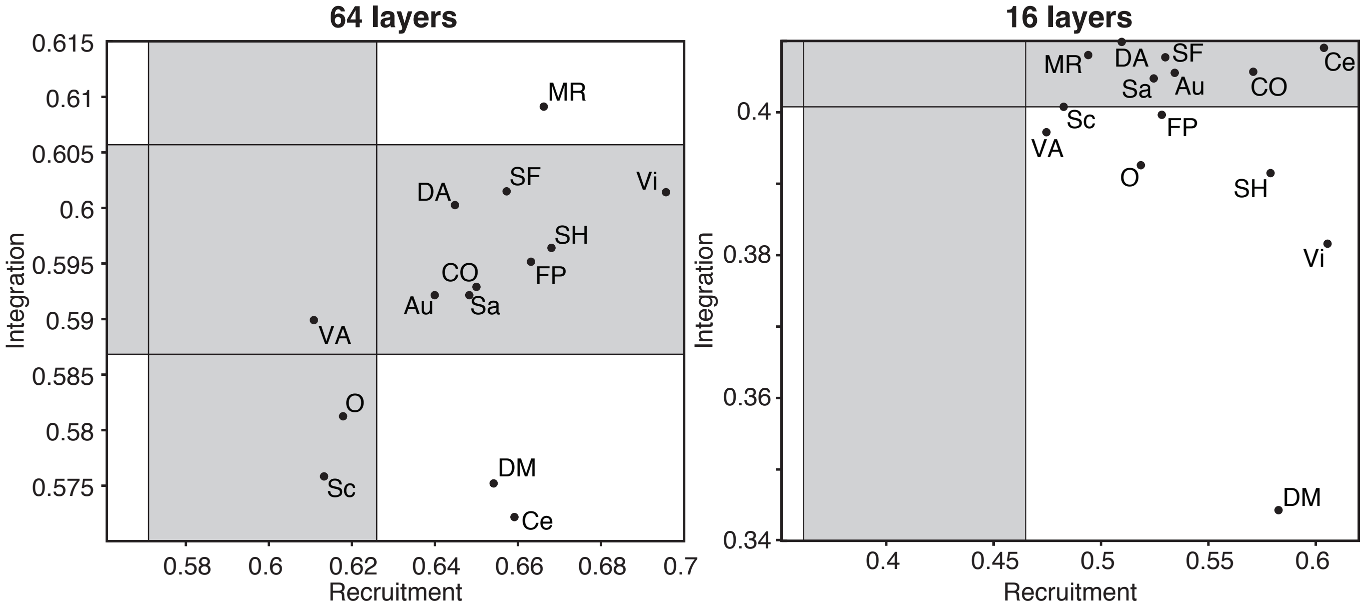

Figure S6: **Examining network organization over longer time windows**
(A) The functional cartography during task execution utilizing a 64-layer network with each layer corresponding to an individual task (see main manuscript). Each system is represented in a position defined by its average recruitment and integration coefficients. Shaded areas – defined by a null model as in panel *(A)* – define the significant regions of the parameter space.
(B) Functional cartography during task execution utilizing a reduced, 16-layer network, with each layer corresponding to a group of four tasks with equal sensory and motor rules.
Abbreviations: VA: Ventral Attention; DA: Dorsal Attention; Sa: Salience; FP: Fronto-Parietal; CO: Cingulo-Opercular; Au: Auditory; SH: Somatomotor Hand; SF: Somatomotor Face; MR: Memory Retrieval; Vi: Visual; Sc: Subcortical; Ce: Cerebellar; DM: Default-Mode; O: Other.

sessions. We estimated pairwise functional connectivity for each task separately, after regressing out (across-trial mean) task-evoked activations and removing the short rest periods between task blocks from each regions time series (see Supplementary Methods).

A search through the γ-ω plane revealed that parameter values of $\gamma = 1.0$ and $omega = 0.45$ yielded a high variability in regional flexibility, accompanied by a relatively stable community architecture (Fig. S7). We note that these values were also consistent with those found to maximize regional flexibility in the 64-task data set. At these parameter values, we calculated the recruitment and integration coefficients of all 14 cognitive systems and we show the resultant HCP-task-based cartography across the 7 tasks in Fig. S8.

It is worth noting several important similarities and differences between the HCP-based task cartography and the PRO-based task cartography. First, in contrast to the multi-block PRO analysis in which tasks were concatenated together, both the HCP-based task cartography and the PRO-based task cartography show cognitive systems occupying 5 different network roles. In fact, in both data sets, cognitive systems occupy the exact same set of roles, including stable integrators, stable loners, unstable loners, stable connectors, and unstable connectors. Interestingly, the 4 roles that are not observed include the 3 that display a lower recruitment coefficient than expected (ephemeral loners, connectors, and integrators), suggesting that in both task sets, the cognitive systems defined in [4] form cohesive temporal units whose regions are consistently allied with other regions in their own system. Perhaps even more interestingly,

half of the cognitive systems display the same role in both the HCP-based task cartography and the PRO-based task cartography: the default mode system is a stable loner, the ventral attention system is an unstable connector, the 'other' system is an unstable loner, and the auditory, dorsal attention, cingulo-opercular, and somatomotor hand systems are stable connectors. The remaining systems are redistributed within the 5 roles common to both task data sets.

Two important and striking differences exist between the HCP-based task cartography and the PRO-based task cartography. First, the average integration coefficient is approximately 1.4 times smaller in the HCP-based task cartography than in the PRO-based task cartography, indicating a decreased probability that regions associate with systems other than their own across the task battery. We speculate that this high degree of temporal consistency in module allegiance is due to the fact that these tasks were created to map out the most salient and independent axes of cognitive function possible. These well-validated tasks thus likely map out modules or cognitive systems that can be consistently and independently activated, requiring little dynamic network integration across the task set. The second striking difference between the two maps is the fact that the range of recruitment coefficients is much smaller in the PRO-based task cartography (range approximately 0.60 to 0.69) than in the HCP-based task cartography (range approximately 0.35 to 0.95). These results suggest the interesting possibility that while the HCP tasks have been chosen to maximize coverage of pre-defined cognitive functions, they do not equally recruit all known cognitive systems. Future work could develop an alternative ontology of cognitive tasks that more equally recruited all cognitive systems across the task set.

Using a Finite Impulse Response Model to Remove Task Effects

In the main text, we removed task-evoked signals from the data by convolving the task regressors with a subject-specific hemodynamic response function (HRF), and using the residuals of this GLM for the esitmation of the functional connectivity between brain regions. One potential concern with this approach is that fitting a unique HRF for the entire brain (even if subject-specific) can leave residual task-evoked signals if the hemodynamics of some regions are very different than the average hemodynamics for the subject. One way to mitigate this confound is to fit an HRF separately for each voxel, using a Finite Impulse Response (FIR) model. Specifically, this model uses a set of delta-function regressors at each time point in a task epoch, allowing for different shapes (amplitude and timing) of HRFs. To assess the relative utility of this method, we applied an FIR model to the PRO data with nine regressors for each of the 12 task rules, for a total of 108 regressors. We used the residuals to calculate the functional connectivity and subsequently estimated the task-based cartography of cognitive systems (Fig. S9). Despite the large number of degrees of freedom used in this model, we only observed very slight differences in the overalll cartography, and the roles of all cognitive systems remained unaltered.

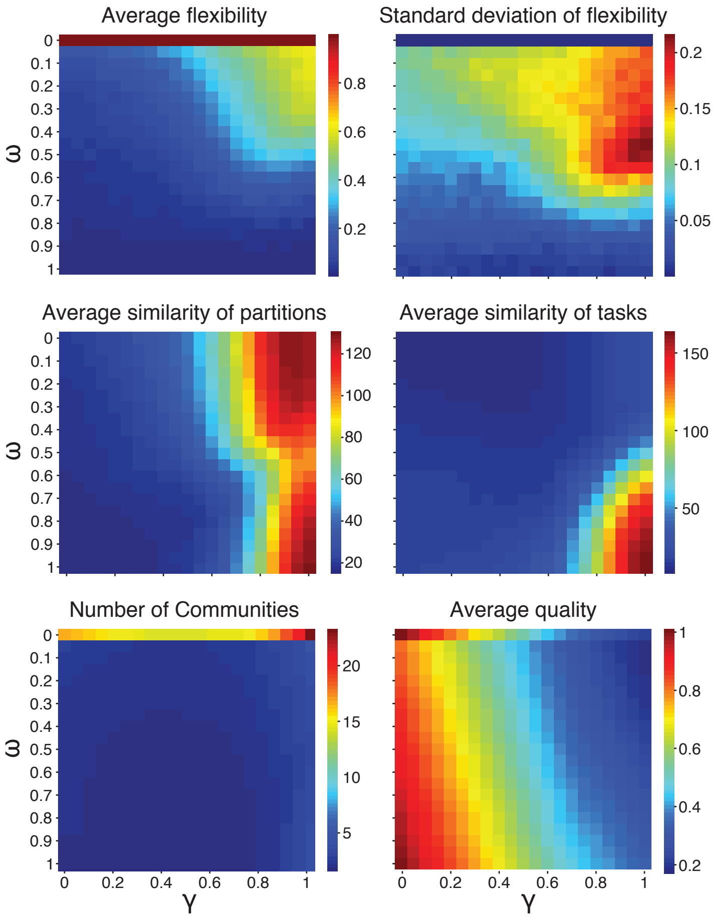

Figure S7: **Grid search over parameter space for the HCP 7-task Data Set.**
Parameter search in the Human Connectome Project data set. Average and standard deviation of the flexibility, average z-score of partition similarity across multilayer modularity maximization and across tasks, average number of communities, and average partition quality Q, calculated for values of the structural resolution parameter (γ) and interslice coupling parameter (ω) that vary between 0 and 1 in intervals of 0.1. We define the optimal combination of parameters as one in which the standard deviation of the flexibility is maximal, with relatively high z-score of partition similarity across multilayer modularity maximization and low z-score of partition similarity across tasks.

References

[1] Murphy, K., Birn, R. M., Handwerker, D. A., Jones, T. B. & Bandettini, P. A. The impact of global signal regression on resting state correlations: are anti-correlated networks introduced? *Neuroimage* **44**, 893–905 (2009).

[2] Fox, M. D., Zhang, D., Snyder, A. Z. & Raichle, M. E. The global signal and observed anticorrelated resting state brain networks. *Journal of neurophysiology* **101**, 3270–3283 (2009).

[3] Satterthwaite, T. D. *et al.* Connectome-wide network analysis of youth with Psychosis-Spectrum symptoms. *Mol Psychiatry* **Epub ahead of print** (2015).

[4] Power, J. D. *et al.* Functional network organization of the human brain. *Neuron* **72**, 665–678 (2011).

[5] Power, J. D., Schlaggar, B. L., Lessov-Schlaggar, C. N. & Petersen, S. E. Evidence for hubs in human functional brain networks. *Neuron* **79**, 798–813 (2013).

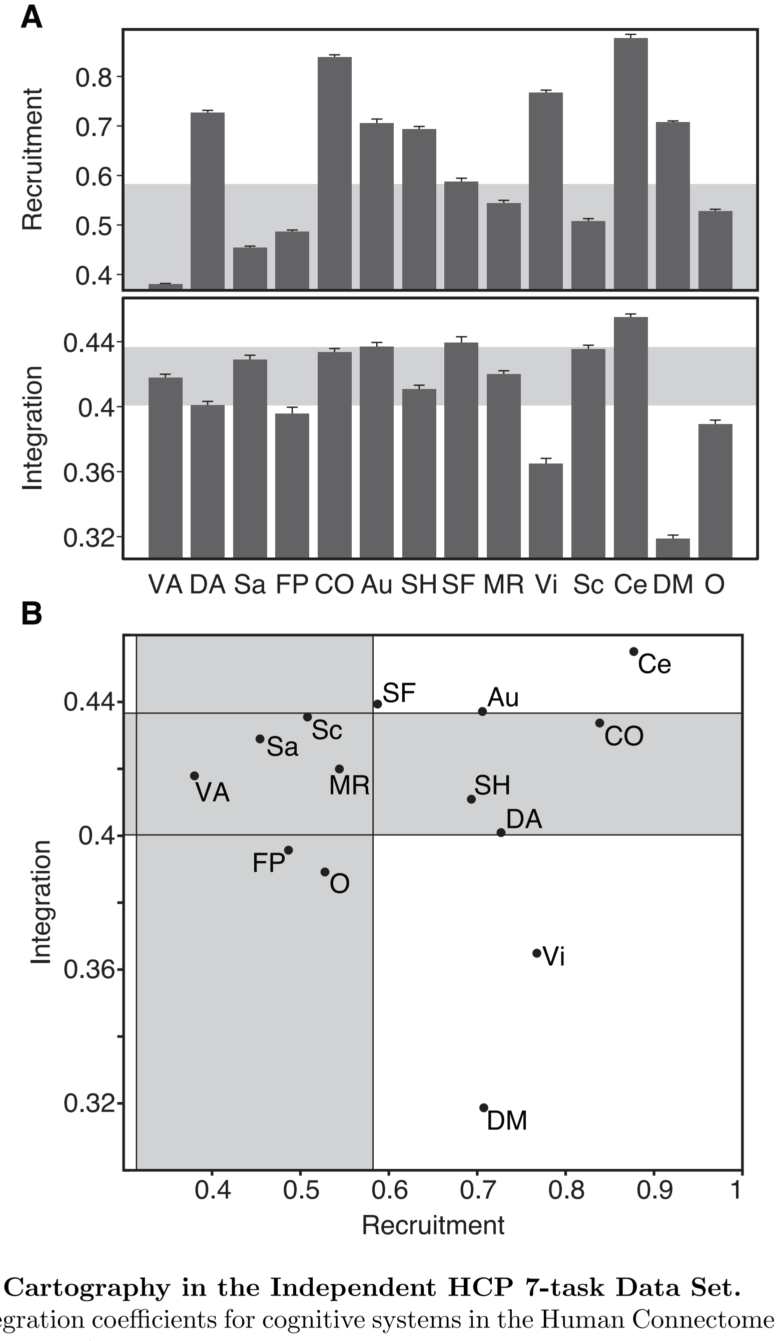

Figure S8: **Task-Based Cartography in the Independent HCP 7-task Data Set.**
(A) Recruitment and integration coefficients for cognitive systems in the Human Connectome Project data set. Shaded areas correspond to the range of values expected by a null model, where each brain region is assigned uniformly at random to a cognitive system. Error bars indicate the standard error of the mean across subjects.
(B) Functional cartography of cognitive systems in the Human Connectome Project data set. Each system is represented in a position defined by its average recruitment and integration coefficients. Shaded areas – defined by a null model as in panel *(A)* – define the significant regions of the parameter space.
Abbreviations: VA: Ventral Attention; DA: Dorsal Attention; Sa: Salience; FP: Fronto-Parietal; CO: Cingulo-Opercular; Au: Auditory; SH: Somatomotor Hand; SF: Somatomotor Face; MR: Memory Retrieval; Vi: Visual; Sc: Subcortical; Ce: Cerebellar; DM: Default-Mode; O: Other.

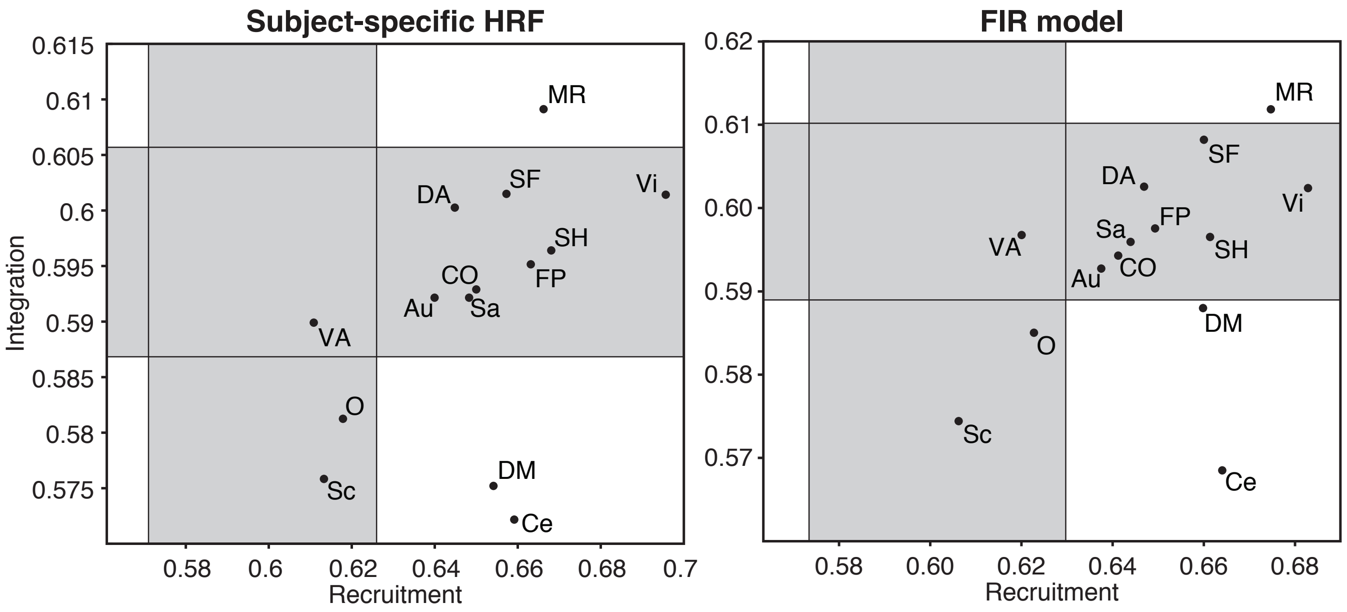

Figure S9: **Removal of Task Effects Through a Finite Impulse Response model.**
(A) The functional cartography during PRO-task execution with a subject-specific HRF estimated and applied to remove residual task-effects prior to the calculation of functional connectivity. We then utilized a 64-layer network with each layer corresponding to the functional connectivity estimated in an individual task (see main manuscript). Each system is represented in a position defined by its average recruitment and integration coefficients. Shaded areas define the significant regions of the parameter space in comparison to a null model.
(B) Functional cartography during task execution with a Finite Impulse Response model applied to remove residual task-effects. This approach aims to remove task effects by modeling the Hemodynamic Response Function (HRF) independently at each brain region and separately for each task, allowing for different shapes (amplitude and timing) of HRFs. The resulting cartography is obtained from the dynamic functional connectivity analysis performed on the residuals of a General Linear Model including nine regressors for each of the 12 task rules, for a total of 108 regressors. Abbreviations: VA: Ventral Attention; DA: Dorsal Attention; Sa: Salience; FP: Fronto-Parietal; CO: Cingulo-Opercular; Au: Auditory; SH: Somatomotor Hand; SF: Somatomotor Face; MR: Memory Retrieval; Vi: Visual; Sc: Subcortical; Ce: Cerebellar; DM: Default-Mode; O: Other.
