# Supplementary material for: A Functional Cartography of Cognitive Systems
Source: PLoS Comput Biol. 2015 Dec 2;11(12):e1004533. doi: 10.1371/journal.pcbi.1004533 (PMC4668064; doi:10.1371/journal.pcbi.1004533)
Supplement: S3 Text — (PDF) [file pcbi.1004533.s003.pdf]

S3 Text. Supplementary Discussion

Parcellation Considerations

In this work, we utilize a coarse-grained parcellation of the brain into 264 functional ROIs [1], which has proven useful in understanding task-based connectivity [2] and its relation to intrinsic connectivity [3], as well as resting state connectivity and its alteration in neurological disorders [4] or change over normative neurodevelopment [5]. Due to its proven sensitivity to changes in functional connectivity across these varied contexts, the 264-node parcellation therefore forms an appropriate choice to assess changes in functional network architecture across task states and our use of this parcellation in this context enables direct comparison of results to prior literature. However, a data-driven assessment of functional regions drawn from voxel-level activity or connectivity and their reconfiguration in whole-brain networks over multiple task states would be an exciting future goal. Reaching this goal will necessitate the development of algorithms to scale multilayer modularity maximization heuristics to larger networks.

Window Length Considerations

In dynamic network analyses, a very important issue is the choice of window length to simultaneously maximize the sensitivity to neurophysiologically driven temporal changes and minimize the sensitivity to spurious temporal changes in network architecture. Recent work makes theoretical arguments in combination with computer simulations to offer 30–60 s as a practical guideline for the choice of window length in dynamic network analyses in the resting state [6]. In contrast, our main analyses are performed on task-based data, where external drivers of activity and connectivity impose added structure in fMRI time series that are non-oscillatory in nature. Furthermore, our results draw on multislice community detection algorithms [7], which are built on multilayer representations of temporal networks [8]. Importantly, these representations contain identity links that connect a node in one time window to itself in other time windows, hard-coding statistical dependence between network states, thereby increasing the statistical reliability of algorithmic detection.

In this context, we chose to employ a window length of 11 TRs (22 s) for two main reasons. First, each novel task is performed in a mini-block that lasted approximately 11 TRs. Thus, an accurate assessment of brain connectivity state during each task requires us to separate the data at approximately these same intervals. Second, prior functional connectivity analyses of these same data have utilized this same window length (11 TRs) and demonstrated the ability to (i) predict task state based on connectivity architecture [2], and (ii) distinguish differences between rest and task states [3]. Thus, we chose to use 11 TR windows for consistency and comparability with previous work, for sensitivity to individual task states, and supported by evidence for meaningful estimates of task-specific functional connectivity patterns. We also note that our choice is consistent with work in other data sets and experimental contexts that has demonstrated relationships between dynamic network statistics calculated in a 12.3 s time window and individual differences in human behavior [9].

In using this small time window, it is important to confirm that the functional connectivity matrices maintain

neurophysiological structure. We therefore applied a static version of the community detection algorithm to the functional networks constructed from the 11 TR windows, to further validate their utility and non-random organization. Specifically, we apply static community detection (optimization of the modularity quality function) on each of the 64 different task windows for each subject to obtain 64×15 partitions of nodes into communities. To validate that these partitions are non-random, we compute the z-score of the Rand coefficient between each data-derived partition and the *a priori* partition of nodes into systems published in [1]. We observe that the partitions derived from the 11 TR windows are significantly similar to the *a priori* partition of nodes into systems: mean $z = 8.89$, corresponding to a p-value of less than 0.00001. In contrast, the average z-score of the Rand coefficient between the *a priori* partition of nodes into systems and a random scrambling of node assignments to data-driven communities was 0.0063. These new results suggest that the 11 TR windows are sensitive to neurophysiologically meaningful network structure.

These results complement our initial evidence for neurophysiological sensitivity drawn from the structure of the module allegiance matrix. In particular, the fact that we observe non-random structure in the module allegiance matrix is clear evidence that partitions in individual time windows do not display random organization. Furthermore, system roles were defined with respect to a null model that used the same window length but permuted system assignments across nodes; if partitions were randomly organized, no cognitive system would exhibit a role different than the null model. Collectively, these results support the use of the 11 TR time window in the analyses reported in this study.

Considerations in System Nomenclature

The system definitions that we use here are based on network community structure in resting state fMRI data acquired from 106 subjects [1]: each network community maps to one of these cognitive systems. Importantly, this mapping of communities to cognitive systems is not a new concept, but instead builds on prior work in functional connectivity data published as early as 2005 [10] and in structural connectivity data published as early as 2008 [11]. These initial papers as well as the work of Power et al. (2011) [1] join a growing literature demonstrating that densely connected brain regions tend to be areas implicated in similar cognitive processes.

However, it remains an open question whether the exact mapping from [1], which was created from resting state data acquired in a different subject cohort, is directly applicable to the task-based data acquired from our subject cohort. To address this question, we performed static community detection on the functional connectivity matrices extracted from each subject and each of the 64 tasks. We then asked whether the network communities that we observed in our data were statistically similar to the systems identified in [1]: that is, did the data-driven partition of nodes into communities in the multi-task data map significantly onto the Power et al. partition of nodes into systems? To quantify the statistical significance of partition similarity, we used the z-score of the Rand coefficient [12], a measure of partition similarity used to examine community architecture in network ensembles. We observed that the partitions obtained from the application of a static modularity maximization to the functional connectivity matrices were significantly similar to the Power et al. partition of nodes into systems (average z-score of the Rand

coefficient was 8.89, corresponding to a p-value of less than 0.00001). In contrast, the average z-score of the Rand coefficient between the Power et al. partition of nodes into systems and a random scrambling of node assignments to data-driven communities was 0.0063, indicating that the randomly permuted partitions were not significantly similar to the Power et al. partition. These results indicate that the partition of nodes into systems reported in [1] and utilized in our analytic pipeline is consistent with the community structure estimated directly from the multi-task data, supporting its use here.

References

[1] Power, J. D. *et al.* Functional network organization of the human brain. *Neuron* **72**, 665–678 (2011).

[2] Cole, M. W. *et al.* Multi-task connectivity reveals flexible hubs for adaptive task control. *Nat Neurosci* **16**, 1348–1355 (2013).

[3] Cole, M. W. *et al.* Intrinsic and task-evoked network architectures of the human brain. *Neuron* **83**, 238–251 (2014).

[4] Cheng, L. *et al.* Reorganization of functional brain networks during the recovery of stroke: a functional MRI study. *Conf Proc IEEE Eng Med Biol Soc* **2012**, 4132–4135 (2012).

[5] Satterthwaite, T. D. *et al.* Linked sex differences in cognition and functional connectivity in youth. *Cereb Cortex* **Epub ahead of print** (2014).

[6] Leonardi, N. & Van De Ville, D. On spurious and real fluctuations of dynamic functional connectivity during rest. *Neuroimage* **104**, 430–436 (2015).

[7] Mucha, P. J., Richardson, T., Macon, K., Porter, M. A. & Onnela, J. P. Community structure in time-dependent, multiscale, and multiplex networks. *Science* **328**, 876–878 (2010).

[8] Holme, P. & Saramäki, J. Temporal networks. *Phys. Rep.* **519**, 97–125 (2011).

[9] Thompson, G. J. *et al.* Short-time windows of correlation between large-scale functional brain networks predict vigilance intraindividually and interindividually. *Hum Brain Mapp* **34**, 3280–3298 (2013).

[10] Salvador, R. *et al.* Neurophysiological architecture of functional magnetic resonance images of human brain. *Cereb Cortex* **15**, 1332–1342 (2005).

[11] Chen, Z. J., He, Y., Rosa-Neto, P., Germann, J. & Evans, A. C. Revealing modular architecture of human brain structural networks by using cortical thickness from MRI. *Cereb Cortex* **18**, 2374–2381 (2008).

[12] Traud, A. L., Kelsic, E. D., Mucha, P. J. & Porter, M. A. Comparing community structure to characteristics in online collegiate social networks. *SIAM Review* **53**, 526–543 (2011).
