# Supplementary material for: A Functional Cartography of Cognitive Systems
Source: PLoS Comput Biol. 2015 Dec 2;11(12):e1004533. doi: 10.1371/journal.pcbi.1004533 (PMC4668064; doi:10.1371/journal.pcbi.1004533)
Supplement: S1 Table — List of abbreviations for cognitive systems (PDF) [file pcbi.1004533.s004.pdf]

S1 Table. Abbreviations.

Table in S1 Table displays a list of abbreviations used for cognitive systems.

**Abbreviation**	**System**
VA	Ventral Attention
DA	Dorsal Attention
Sa	Salience
FP	Fronto-Parietal
CO	Cingulo-Opercular
Au	Auditory
SH	Somatomotor Hand
SF	Somatomotor Face
MR	Memory Retrieval
Vi	Visual
Sc	Cubcortical
Ce	Cerebellar
DM	Default-Mode
O	Other

Table 1: List of abbreviations for cognitive systems
