# Supplementary figures and images for: A Functional Cartography of Cognitive Systems
Source: PLoS Comput Biol. 2015 Dec 2;11(12):e1004533. doi: 10.1371/journal.pcbi.1004533 (PMC4668064; doi:10.1371/journal.pcbi.1004533)

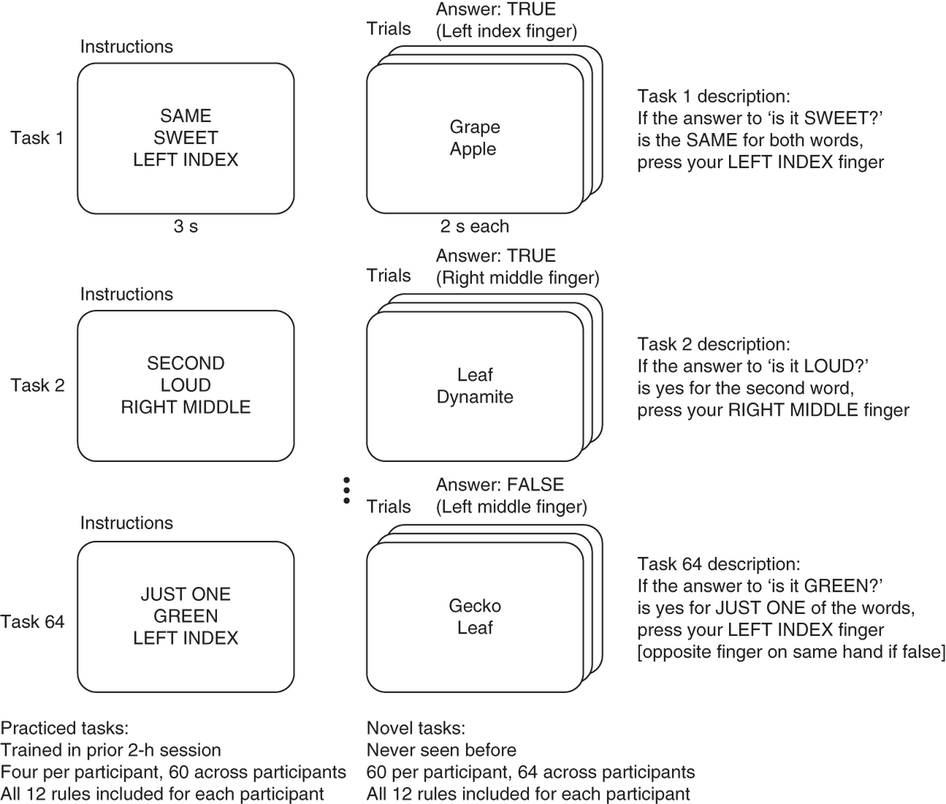

Supplement: S1 Fig — The permuted rule operations (PRO) procedure allowed for behavioral tasks to be created by uniquely combining rules such that the same stimuli could elicit a distinct set of cognitive operations across distinct tasks. Each one of the 64 tasks combine one of four possible logical decision rules, one of four possible sensory semantic rules, and one of four possible motor response rules. Out of the 64 possible tasks, subjects practiced four in a behavioral session prior to the neuroimaging session and the remaining 60 tasks were practiced for the first time during the scan. Participants were over 90% accurate for both novel and practiced tasks. (JPG) [file pcbi.1004533.s005.jpg]

**A**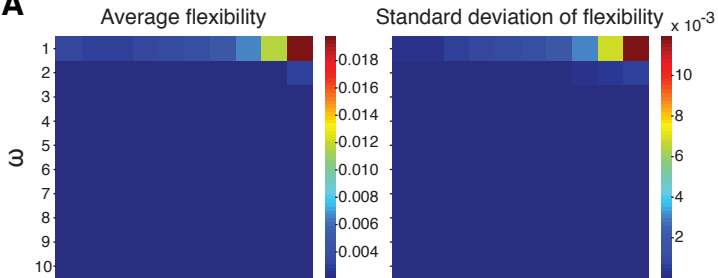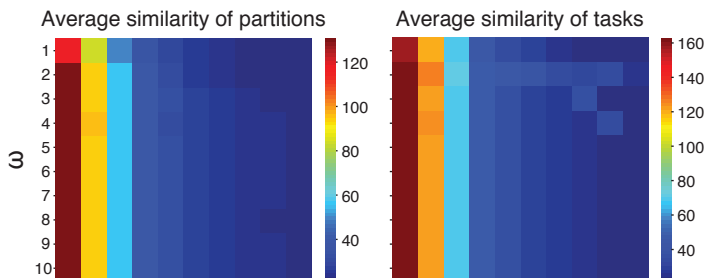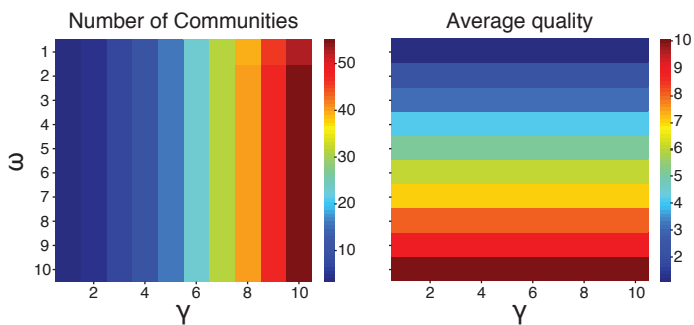**B**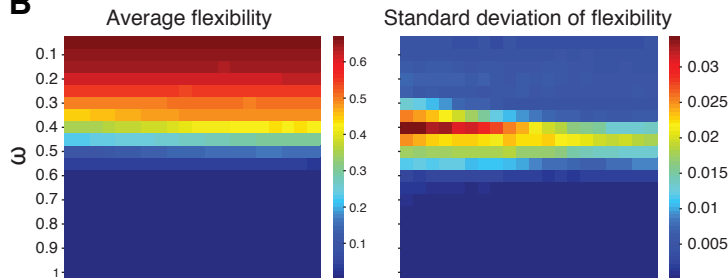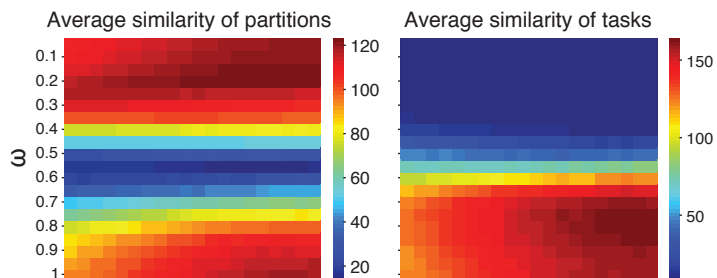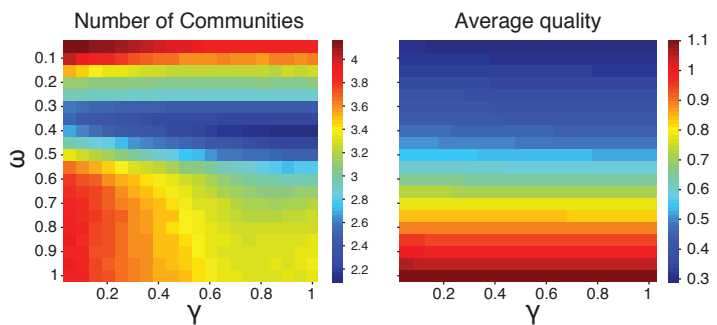

Supplement: S2 Fig — (A) Average and standard deviation of the flexibility coefficient, average community structure similarity across partitions and across tasks, average number of communities, and average partition quality, calculated for structural resolution parameters (γ) and interslice coupling parameters (ω) varying between 1 and 10 in intervals of 1.0. The optimal combination of parameters is one in which the standard deviation of the flexibility coefficient is maximum, with relatively high community structure similarity across partitions and low community structure similarity across tasks. (B) Average and standard deviation of the flexibility coefficient, average community structure similarity across partitions and across tasks, average number of communities, and average partition quality, calculated for structural resolution parameter (γ) and interslice coupling parameter (ω) varying between 0.0 and 1.0 in intervals of 0.05. (PDF) [file pcbi.1004533.s006.pdf]

$\omega=0.45, \gamma=1.0$ 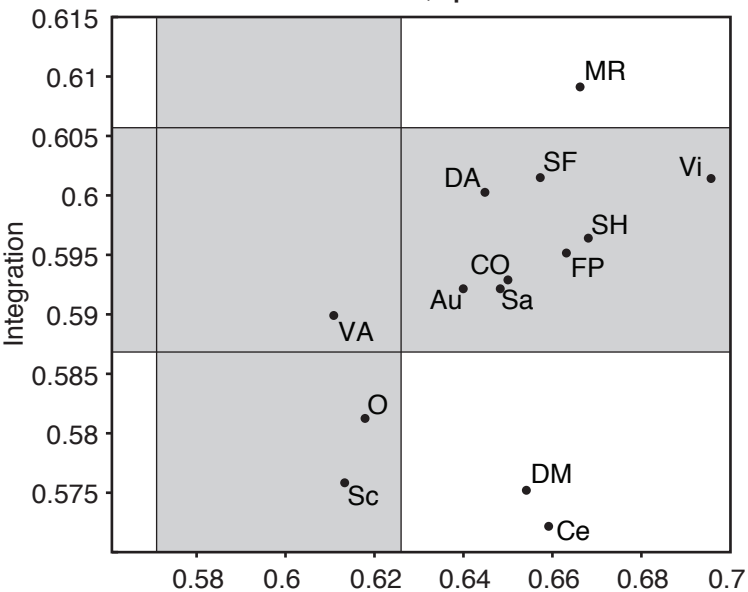 $\omega=0.45, \gamma=0.95$ 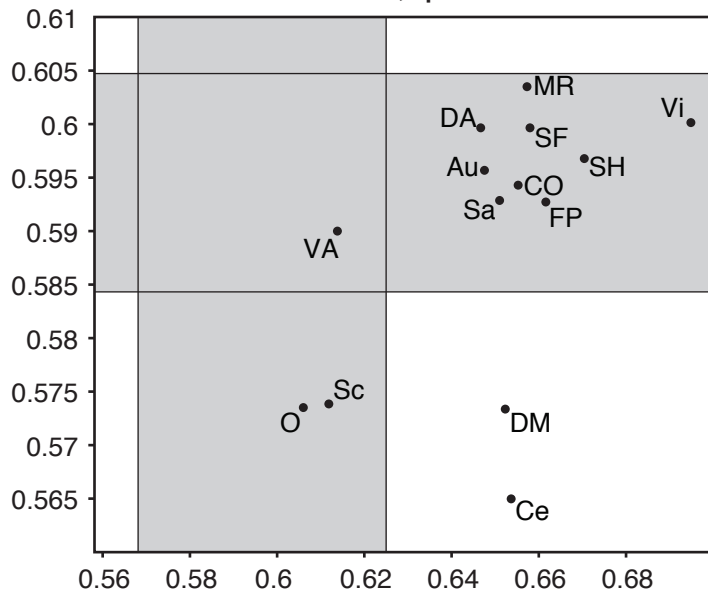 $\omega=0.44, \gamma=1.0$ 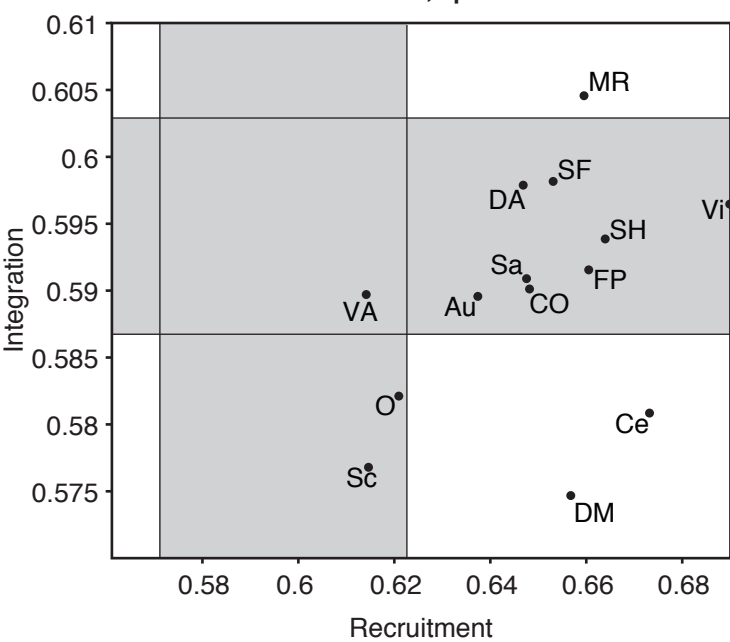 $\omega=0.46, \gamma=1.0$ 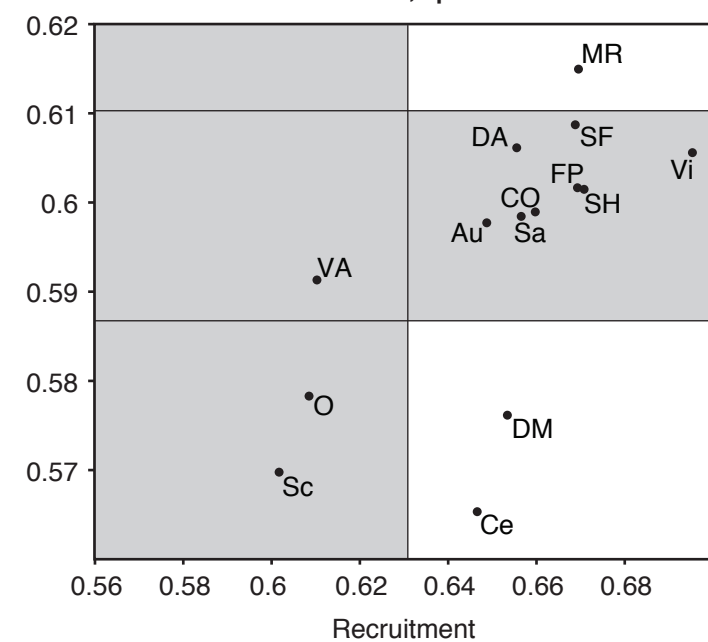

Supplement: S3 Fig — Two-dimensional cartography representations of 14 cognitive systems in the integration-recruitment plane for the following parameter pair choices: (ω = 0.45, γ = 1), top left; (ω = 0.45, γ = 0.95), top right; (ω = 0.44, γ = 1.0), bottom left; (ω = 0.46, γ = 1.0), bottom right. (PDF) [file pcbi.1004533.s007.pdf]

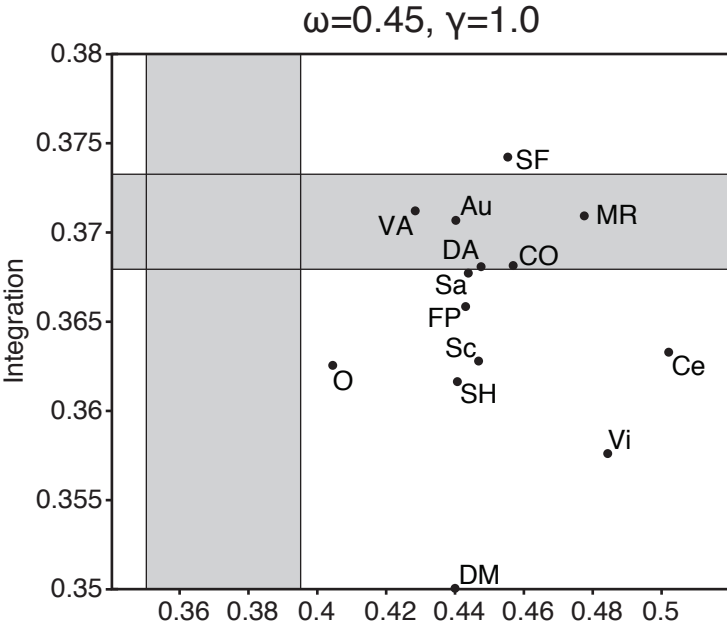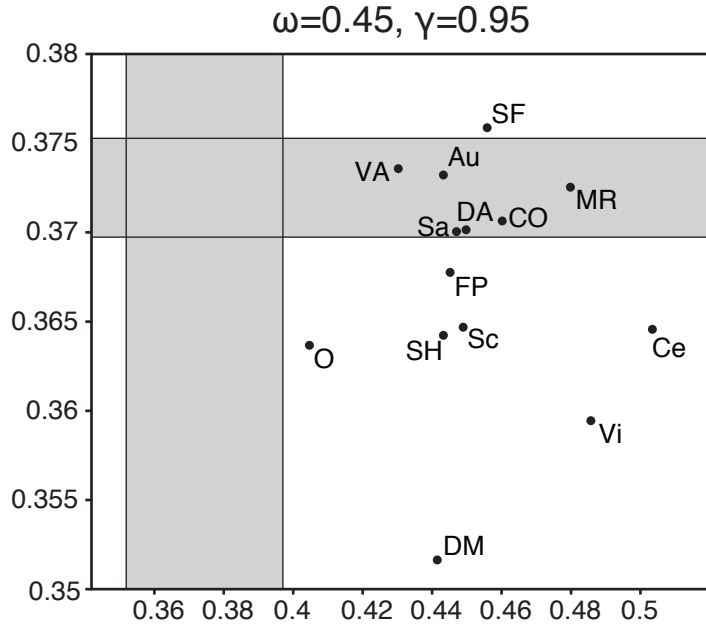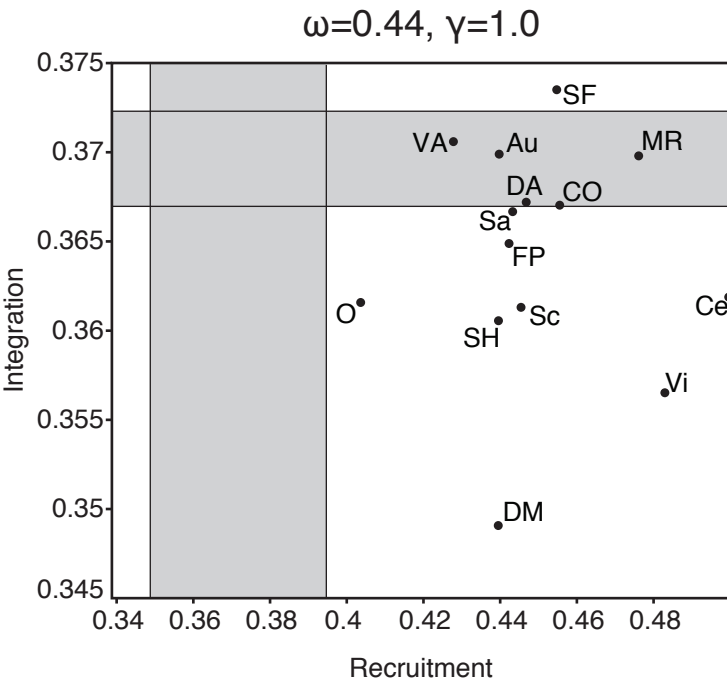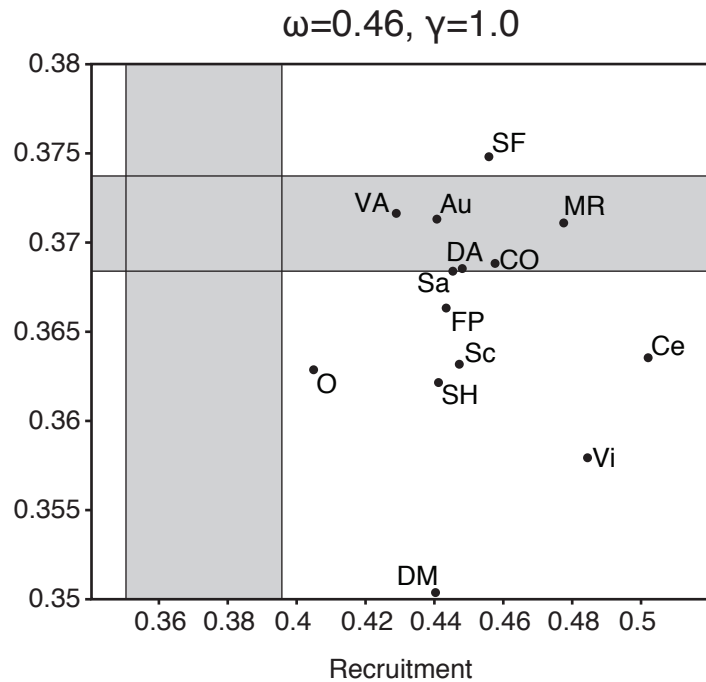

Supplement: S4 Fig — Two-dimensional cartography representations of 14 cognitive systems in the integration-recruitment plane for the following parameter pair choices: (ω = 0.45, γ = 1), top left; (ω = 0.45, γ = 0.95), top right; (ω = 0.44, γ = 1.0), bottom left; (ω = 0.46, γ = 1.0), bottom right. (PDF) [file pcbi.1004533.s008.pdf]

**A**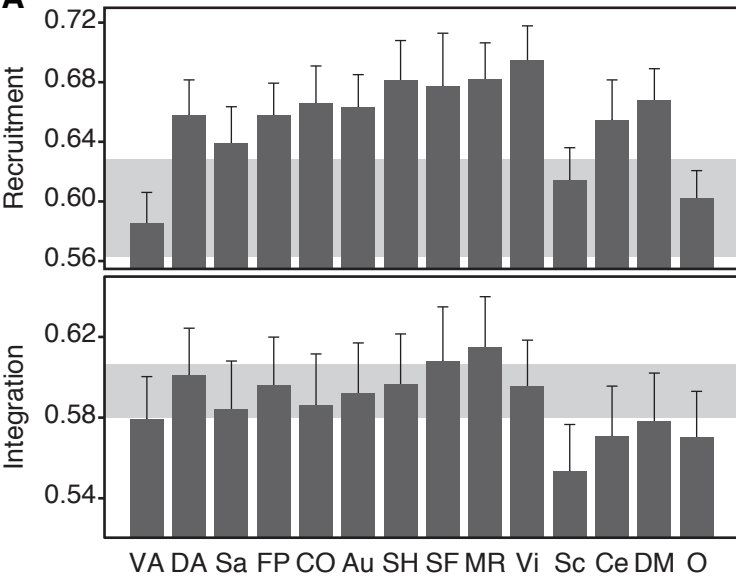**B**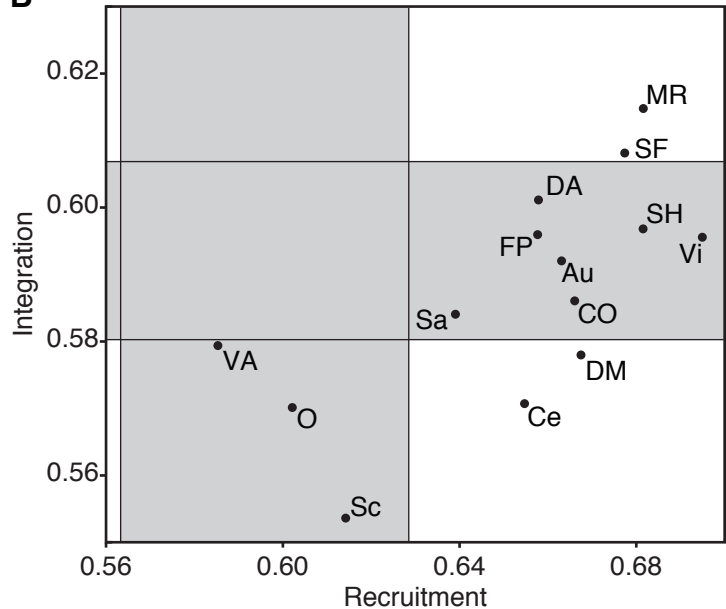

Supplement: S5 Fig — (A) Recruitment and integration coefficients for cognitive systems in the resting state. Shaded areas correspond to the range of values expected by a null model, where each brain region is assigned uniformly at random to a cognitive system. Error bars indicate the standard error of the mean across subjects. (B) Functional cartography of cognitive systems in the resting state. Each system is represented in a position defined by its average recruitment and integration coefficients. Shaded areas—defined by a null model as in panel (A)—define the significant regions of the parameter space. Abbreviations: VA: Ventral Attention; DA: Dorsal Attention; Sa: Salience; FP: Fronto-Parietal; CO: Cingulo-Opercular; Au: Auditory; SH: Somatomotor Hand; SF: Somatomotor Face; MR: Memory Retrieval; Vi: Visual; Sc: Subcortical; Ce: Cerebellar; DM: Default-Mode; O: Other. (PDF) [file pcbi.1004533.s009.pdf]

64 layers

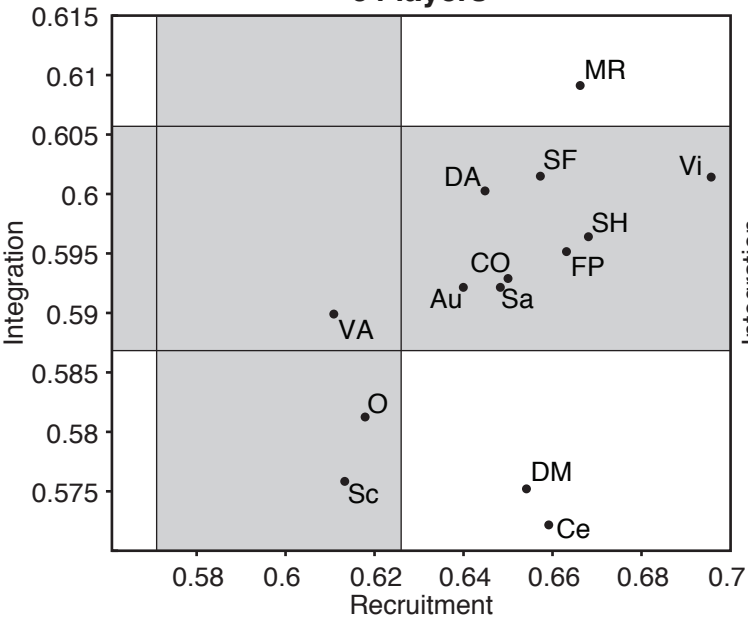

16 layers

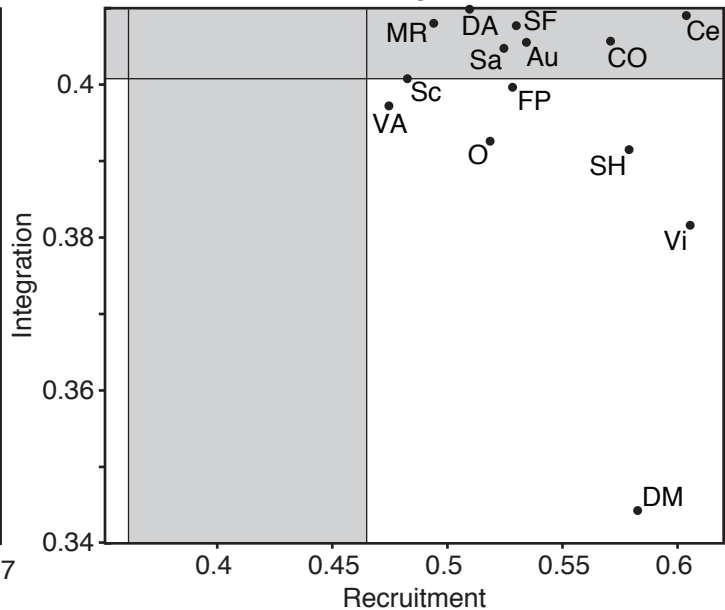

Supplement: S6 Fig — (A) The functional cartography during task execution utilizing a 64-layer network with each layer corresponding to an individual task (see main manuscript). Each system is represented in a position defined by its average recruitment and integration coeffcients. Shaded areas—defined by a null model as in panel (A)—define the significant regions of the parameter space. (B) Functional cartography during task execution utilizing a reduced, 16-layer network, with each layer corresponding to a group of four tasks with equal sensory and motor rules. Abbreviations: VA: Ventral Attention; DA: Dorsal Attention; Sa: Salience; FP: Fronto-Parietal; CO: Cingulo-Opercular; Au: Auditory; SH: Somatomotor Hand; SF: Somatomotor Face; MR: Memory Retrieval; Vi: Visual; Sc: Subcortical; Ce: Cerebellar; DM: Default-Mode; O: Other. (PDF) [file pcbi.1004533.s010.pdf]

**A**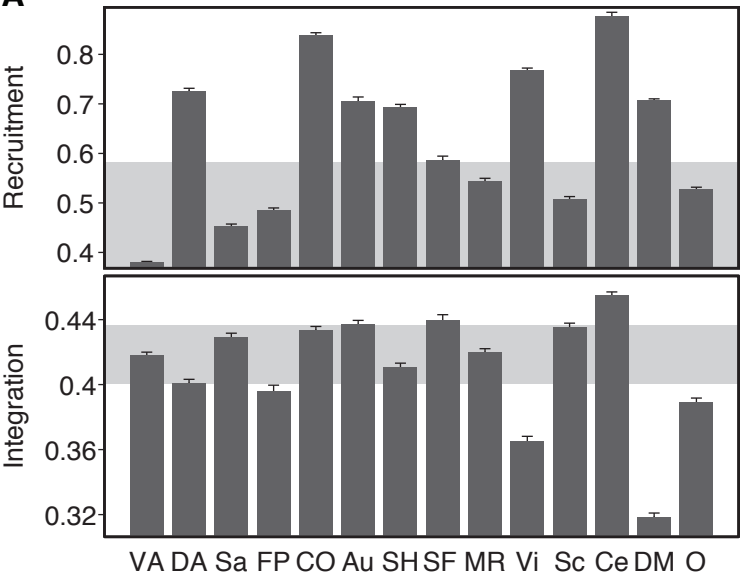**B**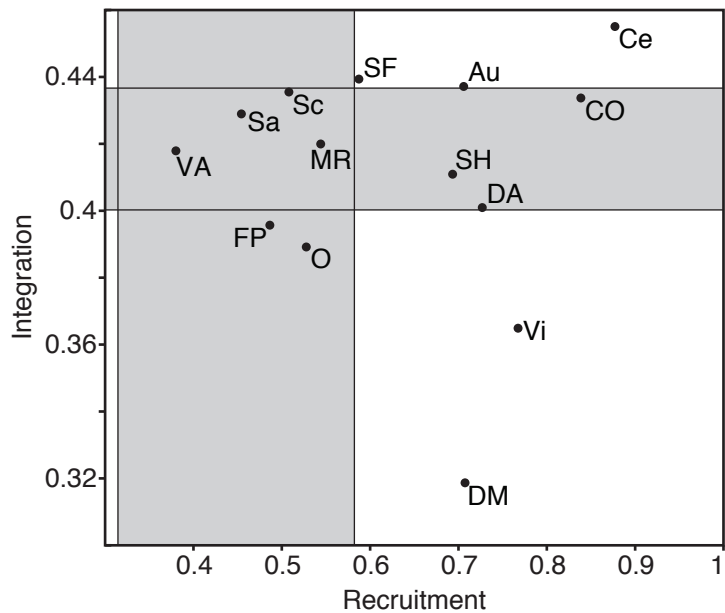

Supplement: S8 Fig — (A) Recruitment and integration coefficients for cognitive systems in the Human Connectome Project data set. Shaded areas correspond to the range of values expected by a null model, where each brain region is assigned uniformly at random to a cognitive system. Error bars indicate the standard error of the mean across subjects. (B) Functional cartography of cognitive systems in the Human Connectome Project data set. Each system is represented in a position defined by its average recruitment and integration coefficients. Shaded areas—defined by a null model as in panel (A)—define the significant regions of the parameter space. Abbreviations: VA: Ventral Attention; DA: Dorsal Attention; Sa: Salience; FP: Fronto-Parietal; CO: Cingulo-Opercular; Au: Auditory; SH: Somatomotor Hand; SF: Somatomotor Face; MR: Memory Retrieval; Vi: Visual; Sc: Subcortical; Ce: Cerebellar; DM: Default-Mode; O: Other. (PDF) [file pcbi.1004533.s012.pdf]

**Subject-specific HRF**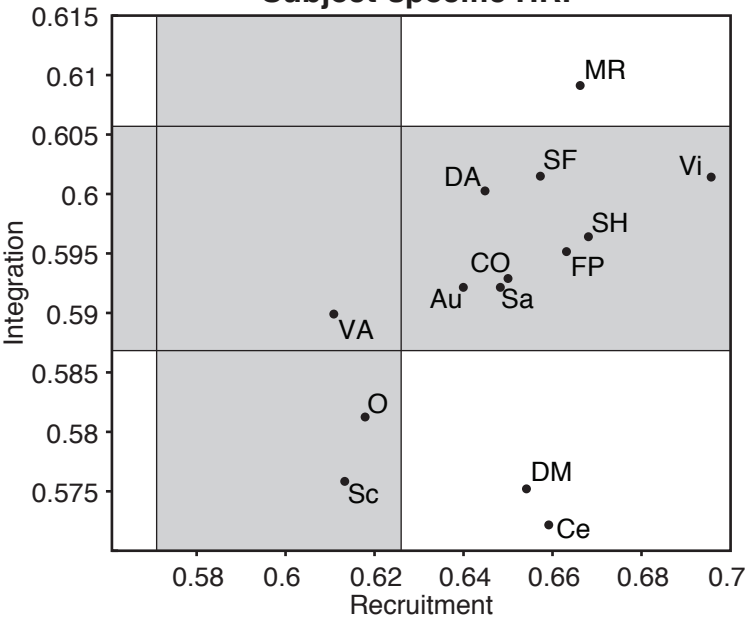**FIR model**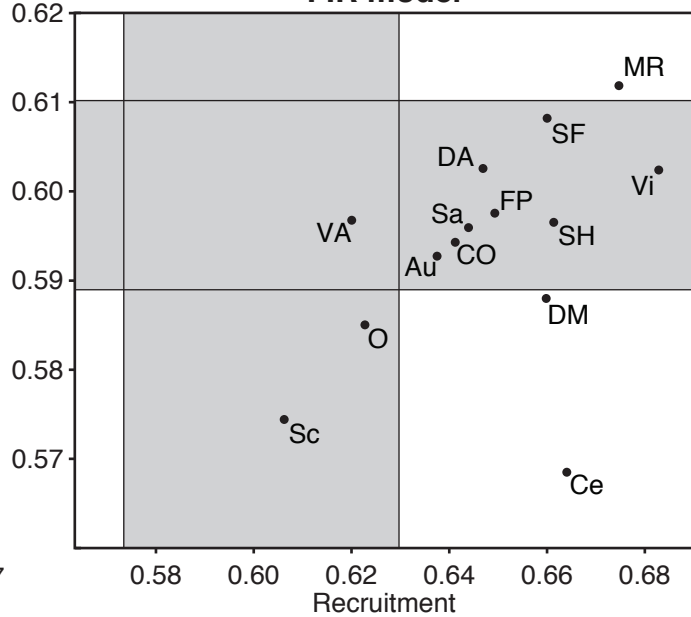

Supplement: S9 Fig — (A) The functional cartography during PRO-task execution with a subject-specific HRF estimated and applied to remove residual task-effects prior to the calculation of functional connectivity. We then utilized a 64-layer network with each layer corresponding to the functional connectivity estimated in an individual task (see main manuscript). Each system is represented in a position defined by its average recruitment and integration coefficients. Shaded areas define the significant regions of the parameter space in comparison to a null model. (B) Functional cartography during task execution with a Finite Impulse Response model applied to remove residual task-effects. This approach aims to remove task effects by modeling the Hemodynamic Response Function (HRF) independently at each brain region and separately for each task, allowing for different shapes (amplitude and timing) of HRFs. The resulting cartography is obtained from the dynamic functional connectivity analysis performed on the residuals of a General Linear Model including nine regressors for each of the 12 task rules, for a total of 108 regressors. Abbreviations: VA: Ventral Attention; DA: Dorsal Attention; Sa: Salience; FP: Fronto-Parietal; CO: Cingulo-Opercular; Au: Auditory; SH: Somatomotor Hand; SF: Somatomotor Face; MR: Memory Retrieval; Vi: Visual; Sc: Subcortical; Ce: Cerebellar; DM: Default-Mode; O: Other. (PDF) [file pcbi.1004533.s013.pdf]
