# Supplementary material for: A Functional Cartography of Cognitive Systems
Source: PLoS Comput Biol. 2015 Dec 2;11(12):e1004533. doi: 10.1371/journal.pcbi.1004533 (PMC4668064; doi:10.1371/journal.pcbi.1004533)
Supplement: S7 Fig — Parameter search in the Human Connectome Project data set. Average and standard deviation of the flexibility, average z-score of partition similarity across multilayer modularity maximization and across tasks, average number of communities, and average partition quality Q, calculated for values of the structural resolution parameter (γ) and interslice coupling parameter (ω) that vary between 0 and 1 in intervals of 0.1. We define the optimal combination of parameters as one in which the standard deviation of the flexibility is maximal, with relatively high z-score of partition similarity across multilayer modularity maximization and low z-score of partition similarity across tasks. (PDF) [file pcbi.1004533.s011.pdf]

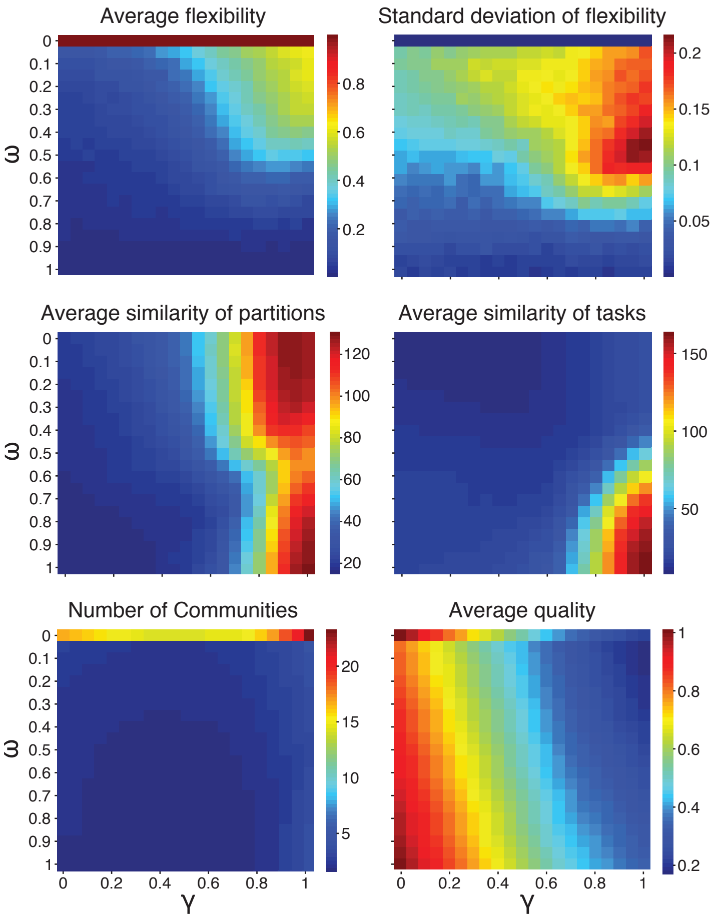
Average flexibility
Standard deviation of flexibility
Average similarity of partitions
Average similarity of tasks
Number of Communities
Average quality
ω
γ
0
0.1
0.2
0.3
0.4
0.5
0.6
0.7
0.8
0.9
1
0.2
0.4
0.6
0.8
0.05
0.1
0.15
0.2
20
40
60
80
100
120
50
100
150
5
10
15
20
0.2
0.4
0.6
0.8
1
